# Development of empirical anti-inflammatory diet index: a cross-sectional study

**DOI:** 10.1186/s12937-025-01165-x

**Published:** 2025-06-23

**Authors:** Joanna Kaluza, Lisa Hellerström, Daniel Kaluza, Abbas Chabok, Agneta Åkesson, Karl Michaëlsson, Alicja Wolk

**Affiliations:** 1 Unit of Cardiovascular and Nutritional Epidemiology, Institute of Environmental Medicine, Karolinska Institutet, Stockholm, SE-171 77 Sweden; 2 Department of Human Nutrition, Warsaw University of Life Sciences – SGGW, Warsaw, 02-776 Poland; 3 Centre for Clinical Research, Västerås Hospital, Uppsala University, Västerås, 721 89 Sweden; 4 Institute of Informatics, University of Warsaw, Warsaw, 02-097 Poland; 5 Division of Surgery, Danderyd University Hospital, Stockholm, 182 88 Sweden; 6 Medical Epidemiology, Department of Surgical Sciences, Uppsala University, Uppsala, SE-751 85 Sweden

**Keywords:** Anti-inflammatory diet index, C-reactive protein, Food, Inflammation

## Abstract

**Background:**

There is evidence that some foods and dietary patterns may influence low-grade inflammation status. We aimed to develop a user-friendly empirical Anti-inflammatory Diet Index (eADI) that predicts low-grade chronic inflammation.

**Methods:**

In this cross-sectional study of 4,432 men (aged 74 ± 6 years) from the Cohort of Swedish Men-Clinical, inflammatory status was assessed by high-sensitivity C-reactive protein (hsCRP), interleukin 6 (IL-6), tumor necrosis factor receptor 1 (TNF-R1), and tumor necrosis factor receptor 2 (TNF-R2). Dietary intake was assessed using a food frequency questionnaire. The eADI was developed in a randomly chosen Discovery group (*n* = 2,216) using a 10-fold feature selection with filtering (based on Lasso regression) to select food groups most correlated with inflammatory biomarkers. From the selected foods, the eADI was then constructed based on summed scores of the consumption tertiles (corresponding to 0, 0.5, and 1 point). Next, in the Replication group (*n* = 2,216), the association of eADI with inflammatory biomarkers was examined using multivariable-adjusted linear regression models.

**Results:**

eADI-17 included 17 food groups (11 with anti-inflammatory, 6 with pro-inflammatory potential). In the Replication group, the median of eADI-17 was 9 (range: 2–16) scores and the Spearman correlation coefficients for eADI-17 vs. hsCRP, IL-6, TNF-R1, and TNF-R2 were -0.17, -0.23, -0.28, and -0.26, respectively. Each increment by 4.5-point eADI-17 (2 SD) was associated with concentrations that were 12% lower for hsCRP, 6% lower for IL-6, 8% lower for TNF-R1, and 9% lower for TNF-R2. These results obtained for the Replication group were robust as they were essentially the same as those of the Discovery group.

**Conclusions:**

The eADI-17 is a validated, robust and user-friendly anti-inflammatory diet index developed to predict low-grade chronic inflammation. This index has the potential to further refine future dietary guidelines and to be used in personalized nutrition. However, its predictive validity should be further evaluated in diverse populations.

**Supplementary Information:**

The online version contains supplementary material available at 10.1186/s12937-025-01165-x.

## Introduction

Low-grade inflammation is characterized by chronic systemic immune system activation [1, 2] without apparent triggers such as acute infection or tissue damage [1]. It has been established that low-grade inflammation promotes cardiovascular disease (CVD) [3, 4], type 2 diabetes [5], osteoporosis [6, 7], and cancers [4, 8]. In this context, identifying foods and dietary patterns with anti- or pro-inflammatory potential is of public health interest [9–11].

A few dietary indices have been developed to assess the anti- or proinflammatory potential of diet. However, their practical use remains limited due to the lack of presenting a simple method for scoring of individuals. In a clinical setting for personalized dietary advice, neither the literature-derived Dietary Inflammatory Index (DII) [9] nor the Empirical Dietary Inflammatory Index (EDII) [10] allows for the identification of individuals at increased risk of diet-related low-grade inflammation. The empirically developed Anti-Inflammatory Diet Index (AIDI) [11], for which scoring criteria for an individual diet have been reported, is based on only one inflammatory biomarker (high-sensitivity C-reactive protein, hsCRP), which might be a limitation. Given that the immune system is a complex physiological system, it is unlikely that only one inflammatory biomarker can reliably assess its activity. Therefore, multiple inflammatory biomarkers should be considered to reflect the diverse aspects of the immune response [12].

To address this limitation, we aimed to develop and validate an easy-to-use diet index with clear scoring criteria – the empirical Anti-inflammatory Diet Index (eADI) using multiple inflammatory biomarkers. We hypothesized that the increasing adherence to a diet aligned with the eADI is reflected in lower concentrations of hsCRP, interleukin 6 (IL-6), tumor necrosis factor receptor 1 (TNF-R1), and tumor necrosis factor receptor 2 (TNF-R2). Moreover, eADI also has the potential to reduce inflammation in subgroups with higher inflammatory potential such as old age, smokers, abdominal obesity, or in those with chronic diseases.

## Material & methods

### Study population

**Fig. 1:**
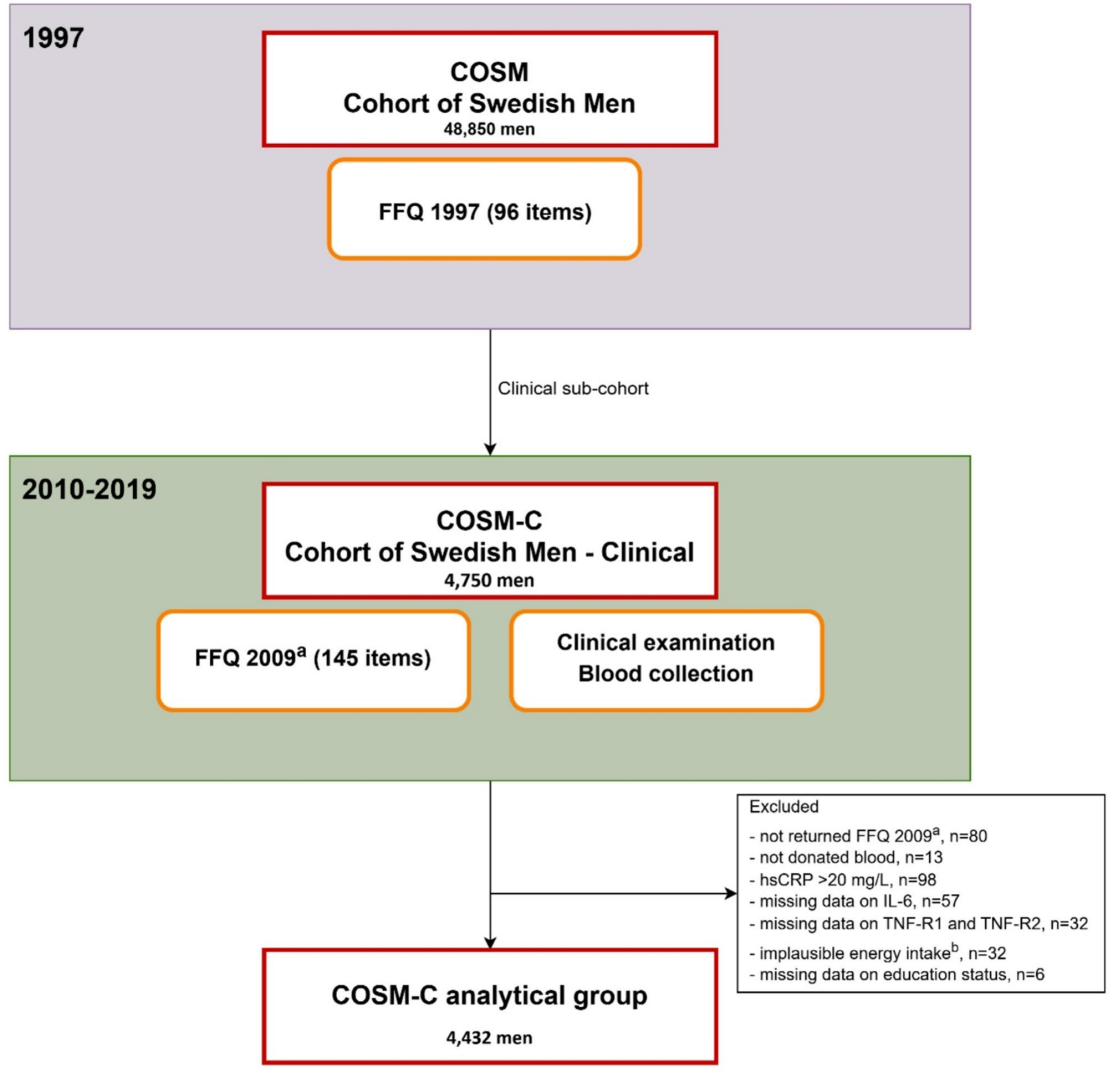
Flowchart of the Cohort of Swedish Men - Clinical (COSM-C). **Legend**: Abbreviations: FFQ, food frequency questionnaire; hsCRP, high-sensitivity C-reactive protein; IL-6, interleukin 6; TNF-R1, tumor necrosis factor receptor 1; TNF-R2, tumor necrosis factor receptor 2. ^a^ FFQ developed in 2009. ^b^ Energy intake ± 3 standard deviations from the mean value for log-transformed energy intake

The Cohort of Swedish Men (COSM) was established in 1997 when all men aged 45–79 from Västmanland and Örebro Counties (central Sweden) were invited to complete a questionnaire on food intake and other lifestyle factors [13]. COSM is part of the Swedish Infrastructure for Medical Population-based Life-course and Environmental Research (SIMPLER), which was established to provide data for research on diet, lifestyle, disease development, and health [14]. Between May 2010 and June 2019 4,750 men from the COSM participated in research reception examinations, including collection of different types of fasting samples, and the COSM comprehensive sub-cohort (COSM^CS^) was established [14, 15]. The flowchart of the COSM^CS^ is presented in Fig. 1. The men were asked to complete the COSM^CS^ lifestyle questionnaire, including a 145-item food frequency questionnaire (FFQ), and a health questionnaire, and to donate blood, urine, fat biopsy, and fecal samples. Among them, 80 men did not return the FFQ, and 13 did not donate blood; thus, 4,657 men were eligible for the present study. Furthermore, we excluded men with hsCRP > 20 mg/L (*n* = 98) because such levels could be due to infection or other intensive inflammatory processes, those with missing data on IL-6 (*n* = 57) or TNF-R1 and TNF-R2 (*n* = 32), and those with missing data on education status (*n* = 6). Moreover, men with implausible total energy intake (i.e., > 3 standard deviations from the mean value on a log scale; *n* = 32) were excluded. After the exclusions, 4,432 participants with hsCRP ≤ 20 mg/L remained for cross-sectional analysis. Thus, the study population included participants with low-grade inflammation and those with a low range of marked CRP elevation [16].

### Collection of dietary data and other lifestyle factors

Diet was assessed using the 145-item FFQ section of the lifestyle questionnaire within one to two months before the clinical examination. The FFQ included questions about commonly consumed food items over the last month. Participants were asked to indicate how often, on average, they had consumed various foods by using one from the eight predefined frequency categories: never/seldom, 1–3 times per month, 1–2 times per week, 3–4 times per week, 5–6 times per week, once a day, 2 times a day, or ≥ 3 times per day. Total energy intake in kcal per day was calculated from the FFQ using four age-specific portion sizes (based on one-week weighted diet records among men aged 45–79 years) together with information on the energy content of specific foods provided by the Swedish National Food Composition Database [17]. The validity of a shorter version FFQ (96 food items) has been confirmed among 248 men using 14 repeated 24-hour recall telephone interviews spread over a whole year. The average Spearman correlation coefficients between energy-adjusted macro- and micronutrient intake assessed by the FFQ and 24-hour recalls were 0.65 and 0.62, respectively [18].

The COSM^CS^ lifestyle questionnaire collected information about physical activity and smoking status, and the health questionnaire collected information about sleep duration. Data about education was extracted from the baseline COSM questionnaire in 1997.

### Clinical examination

At clinical examination, height, weight, and sagittal abdominal measurements were taken using standard methods. Body mass index (BMI) was calculated by dividing the weight in kilograms by the square of the height in meters.

The weighted Charlson’s comorbidity index (CCI) was used to evaluate the men’s general health status at the time of the clinical health examination [19, 20]. Information about registered diagnoses according to ICD-10 was obtained by linking COSM to the National Patient Register.

By linking to the National Prescribed Drug Register, we obtained information about prescribed drugs related to inflammation during the year before blood collection. The Anatomical Therapeutical Chemical (ATC) Classification System was used to identify antibiotics (ATC codes J01AA-J01XX), statins (ATC codes C10AA and C10BA), non-steroidal anti-inflammatory drugs (NSAIDs; ATC codes M01AA-M01BA), and cortisone (ATC codes M01BA and H02AB-H02BX). Only oral-use drugs within these categories were included.

### Blood collection and inflammatory biomarkers

Four inflammatory biomarkers, hsCRP, IL-6, TNF-R1, and TNF-R2, were used to develop the eADI. These biomarkers are used to assess associations between diet and health status [21, 22], are associated with the development of several diseases, and are among the most commonly used inflammatory markers to examine disease status [21, 23].

Blood samples were collected in the morning following an overnight fast. For lithium-heparin plasma preparation, blood samples were light-protected, and after a delay of 15–20 min at room temperature, the samples were spun in a centrifuge at 1615 g for 11 min at 4 °C. The plasma was frozen in multiple tubes and stored at -80^o^C until analysis.

Plasma hsCRP was measured using an Architect Ci8200 analyzer with high-sensitivity latex enhanced immunonephelometric assay 6K2601 (Abbott Laboratories, Abbott Park, IL). The intraassay coefficients of variation for hsCRP were 5% at 1.4 mg/L and 4% at 80 mg/L. The concentration of hsCRP was log2 transformed and expressed in mg/L.

IL-6 and TNF-α receptors (TNF-R1 and TNF-R2) were determined by panels CVD II and CVD III from Olink Proteomics (Uppsala, Sweden). The biomarker concentrations were presented as normalized protein expression (NPX) with values as arbitrary units (in a log2 scale) provided by Olink proteomics. The coefficient of variation within and between run precision calculated from linearized NPX values (over limits of detection) for IL-6 was 9%, for TNF-R1 was 8–12%, and for TNF-R2 was 8–10%. The Olink NPX Manager software was applied for data acquisition, and one-unit increment in NPX represents an approximate doubling of measured protein concentration.

### Development of the empirical Anti-inflammatory diet index (eADI)

To develop the eADI, the men were randomly categorized into two equal groups – a Discovery group (*n* = 2,216) and a Replication group (*n* = 2,216). The eADI was developed in the Discovery group and then validated in the Replication group.

#### Identification of foods with anti-inflammatory and pro-inflammatory potential

The dietary data was preprocessed before analysis. First, the 145 food items included in the FFQ were grouped into 33 food groups based on similar nutritional properties (Supplementary Table 1S). Next, the four individual inflammatory biomarkers (hsCRP, IL-6, TNF-R1, and TNF-R2) were standardized to a mean of 0 and a standard deviation of 1, because they were later analyzed using Principal Components Analysis (PCA), which is sensitive to variance of data.

**Fig. 2:**
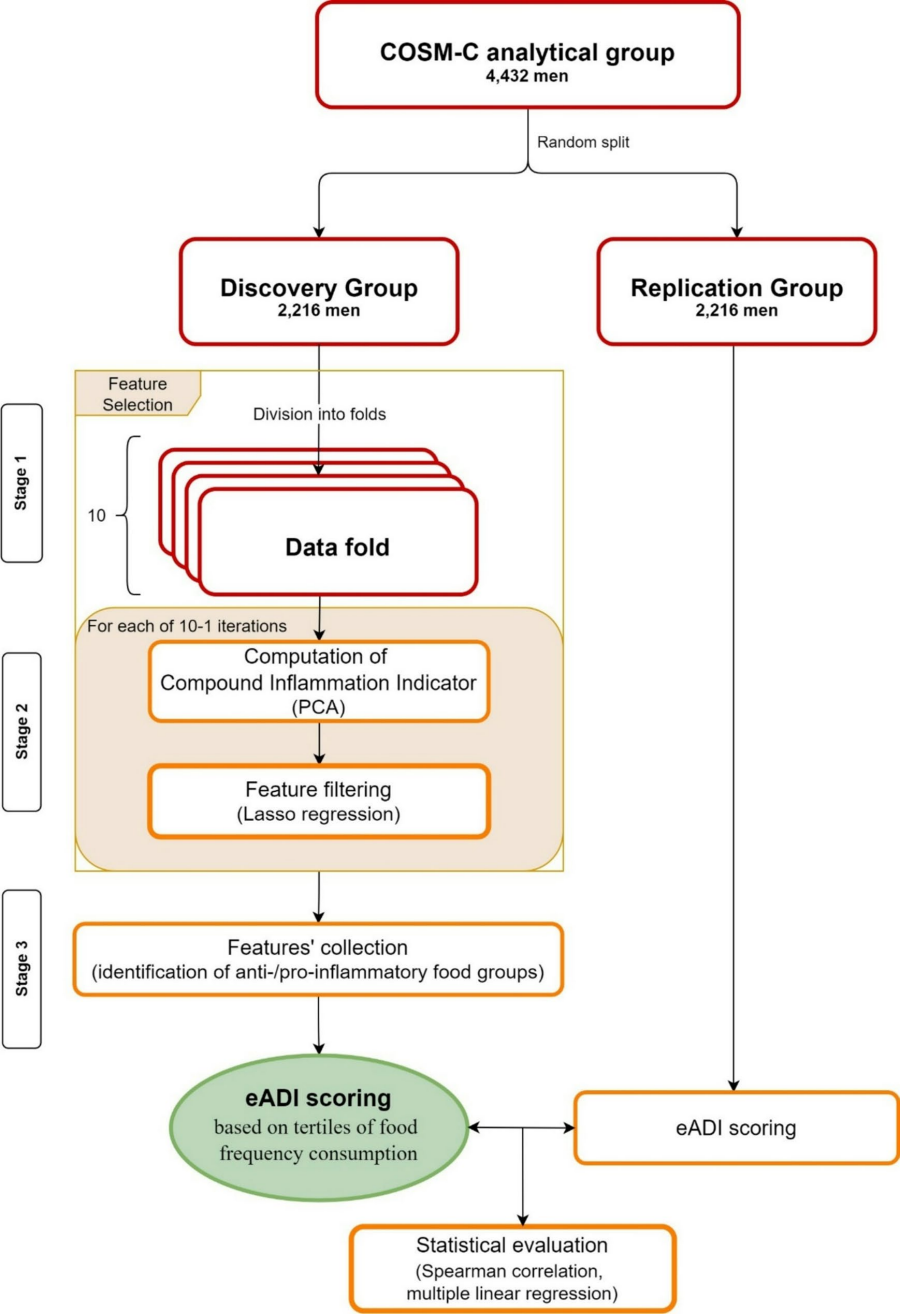
Flowchart of the development of the empirical Anti-inflammatory Diet Index (eADI). **Legend**: Abbreviations: COSM-C, Cohort of Swedish Men - Clinical; PCA, principal component analysis

To identify foods with anti-inflammatory and pro-inflammatory potential, we have used the k-fold cross-validation feature selection method based on Lasso regression. This technique is well-suited for eliminating features that do not significantly contribute to the outcome of interest [24]. Detailed stages of this procedure conducted in the Discovery group are presented in Fig. 2 and Supplementary Table 2S. In the *Data division stage (Stage 1)*, the dataset was randomly split into 10 equally sized, non-overlapping folds. Feature filtering was then conducted across these 10 folds within the Discovery group. *Filtering procedure (Stage 2)* contained two steps: PCA and Lasso regression. This entire procedure was repeated separately for each data fold. PCA was used to identify the main directions of variation of the four inflammatory biomarkers in the Discovery group [25]. Because the first principal component (PC1) was positively correlated with all four pro-inflammatory biomarkers, PC1 values for each participant were used further in Lasso regression denoted as ‘the compound inflammation indicator’. The second principal component (PC2) was positively correlated with hsCRP and IL-6, but negatively correlated with TNF-R1 and TNF-R2. As PC2 was not consistently correlated with all inflammatory biomarkers, it was not included in the feature selection process. Supplementary Fig. 1S visualizes the Spearman correlation coefficients and loadings for PC1 versus PC2. Spearman correlations between the compound inflammatory indicator (PC1) and the individual biomarkers were 0.62 for hsCRP, 0.73 for IL-6, 0.84 for TNF-R1, and 0.82 for TNF-R2; corresponding correlation loadings were 0.41, 0.47, 0.56, and 0.54, respectively. PC1 explained 56% of the variance across these four biomarkers, while PC2 explained 25%.

In the next step of the Filtering procedure, Lasso regression with regularization parameter lambda = 0.5 was used to find the food variables most strongly correlated with the compound inflammation indicator. As Lasso regularization imposes an additional cost on model parameters, it selects only variables with the strongest correlation with the dependent variable (the compound inflammatory indicator) [26].

In the final stage, *Feature collection (stage 3)*, only the most robust features were selected. Features with non-zero coefficients in all or nearly all cross-validation folds were considered reliable predictors. Therefore, to find strong food predictors of inflammation, we established a threshold of non-zero coefficients in at least 9 out of 10 folds. Food groups meeting this criterion in the Lasso regression were selected for inclusion in the eADI (Supplementary Table 3S).

The feature selection method (based on Lasso regression model) was used only to identify food groups most strongly correlated with the inflammatory parameters and to categorize foods into anti- and pro-inflammatory products. This method was not used for any further purposes. A negative coefficient between a selected food and the compound inflammation indicator in the Lasso model indicated the food´s anti-inflammatory properties, while a positive coefficient indicated pro-inflammatory properties. Food products that were not identified as either pro-inflammatory or anti-inflammatory were excluded from the development of the eADI. In Supplementary Table 3S, the mean values and standard deviations of the coefficients for each food group across the cross-validation folds are presented. A predictor was considered strong and stable if it demonstrated the same direction across all folds, had a low mean coefficient value for anti-inflammatory foods or a high mean value for pro-inflammatory foods, and exhibited low standard deviations.

#### eADI points

The contribution of each pro-inflammatory or anti-inflammatory food group to the total eADI score for each participants was calculated based on crude consumption estimates. Points assigned to each food group were based on tertiles of consumption estimated in the Discovery group of the COSM^CS^ population. For anti-inflammatory foods, individuals in the lowest consumption tertile (first tertile) were assigned 0 points, those in the middle tertile (second tertile) received 0.5 points, and those in the highest tertile (third tertile) received 1 point. For pro-inflammatory foods, the scoring was reversed: 1 point was assigned for consumption in the first tertile, 0.5 points for the second tertile, and 0 points for the third tertile. The final eADI score was calculated for each participant by summing the points assigned for all pro-inflammatory and anti-inflammatory food groups. A higher total eADI score indicates a higher anti-inflammatory potential of the diet.

### Statistical analysis

Spearman rank correlation coefficients *(Rs)* were used to estimate the correlation between individual eADI´s components (food groups included in eADI) and the total eADI versus the compound inflammatory indicator (from the PCA) as well as individual hsCRP, IL-6, TNF-1R, and TNF-2R concentrations.

Multivariable-adjusted linear regression models were used to assess the association between the eADI and the single inflammatory biomarkers with the lowest eADI group as a reference. The multivariable models were adjusted for age (years, continuous), education (primary, high school, or university), walking/cycling (< 20, 20–40, 40–60, or > 60 min/day), smoking status (never, former, or current), NSAID use within the last year (no, yes), statin use within the last year (no, yes), cortisone use within the last year (no, yes), antibiotic use in the previous year (no, < 3, 3–6, or 6–12 months from blood collection), an hour of sleep (≤ 7, > 7 days), BMI (< 18.5, 18.5–24.9, 25-29.9, or ≥ 30 kg/m^2^), sagittal measure (cm, quintiles), comorbidity (CCI 0, 1, 2, or ≥ 3 scores), and energy intake (kcal/day, quintiles). Confounders were selected a priori based on factors likely linked to inflammation and covarying with diet quality. Missing data on walking/cycling (0.4%), smoking status (6.3%), hours of sleep (4.8%), BMI (0.2%), and sagittal measurements (0.3%) were modeled as separate missing indicator categories. Tests for linear trends were conducted with the eADI as a continuous variable.

Moreover, we performed subgroup analyses examining the associations between the eADI and hsCRP concentration by hsCRP concentration categories (< 15, < 10, < 5, and < 3 mg/L) to investigate whether the association with anti-inflammatory dietary pattern was dependent on the level of inflammation. Furthermore, we performed the multivariable-adjusted linear regression analyses stratified by age (63–74, 75–84, and 85–94 years old), smoking status (current/ex-smokers, never), sagittal measurement (≤ 22.0, >22.0 cm), and CCI (0, ≥ 1 scores).

All statistical analyses were performed using STATA 13 (StataCrop, College Station, TX); all *P*-values ≤ 0.05 were considered statistically significant and were two-sided.

## Results

### Development of eADI-17

The eADI includes 17 food groups (eADI-17; from originally 33), among which 11 had an anti-inflammatory potential (negatively correlated with the compound inflammatory indicator) and 6 had a pro-inflammatory potential (positively correlated). Lasso regression coefficients for each food group and each fold in the Discovery group are presented in Supplementary Table 3S.

The 11 food groups with anti-inflammatory potential included: vegetables; olive oil and canola oil; nuts; seeds; legumes; wine; muesli; chicken; eggs; wholegrains (bread, macaroni, spaghetti, and rice); and coffee. The 6 food groups with pro-inflammatory potential consisted of: soft-drink beverages; sugar and honey; milk (3% fat); boiled potatoes; processed red meat; and other processed foods (chips, French fries, buns/cakes, biscuits/wafers/rusks, ice cream, jams, ready-to-eat products such as pea soup, pancakes, etc.). Specific foods included in each eADI-17 component are presented in Supplementary Table 1S. The final eADI-17 score constituted the sum of points ascribed to all 17 food components (Table 1).

**Table 1:** Criteria for the scoring consumption of food components included in the empirical Anti-inflammatory diet index (eADI) and percent meeting these criteria in the clinical cohort of Swedish men (Discovery group, *n* = 2,216 men with hsCRP < 20 mg/L)

		Tertiles of consumption			Percent of men who met the criteria		
		0 points	0.5 points	1 point	0 points	0.5 points	1 point
**Foods with anti-inflammatory potential**		***Servings/day (median)***					
	Wholegrains^a^	< 2.5 (1.9)	2.5–4.0 (3.1)	> 4.0 (5.4)	32.9	30.3	36.8
	Vegetables	< 2.2 (1.5)	2.2–3.8 (3.0)	> 3.8 (4.9)	33.1	33.9	33.0
	Coffee	< 2.0 (1.0)	2.0–3.0 (2.0)	> 3.0 (4.0)	18.8	53.7	26.6
	Olive oil and canola oil^b^	0	1	> 1	25.8	29.2	45.0
		***Servings/week (median)***					
	Chicken	0	0.1–0.5 (0.5)	> 0.5 (1.5)	9.0	43.2	47.8
	Eggs	< 1 (0.5)	1.0–2.0 (1.5)	> 2.0 (3.5)	35.4	38.6	26.0
	Legumes	0	0.1–1.0 (0.5)	> 1.0 (1.5)	51.8	35.3	13.0
	Muesli	0	0.1–3.5 (1.5)	> 3.5 (7.0)	56.9	20.9	22.2
	Nuts	0	0.1–1.0 (0.5)	> 1.0 (3.0)	32.7	37.5	29.8
	Seeds	0	0.1–1.0 (0.5)	> 1.0 (7.0)	72.4	5.5	22.1
	Wine	< 0.2 (0)	0.2–1.5 (0.7)	> 1.5 (3.0)	26.4	34.1	39.5
		**0 points**	**0.5 points**	**1 point**	**0 points**	**0.5 points**	**1 point**
**Foods with pro-inflammatory potential**		***Servings/day (median)***					
	Processed meat	> 1.5 (2.2)	0.8–1.5 (1.1)	< 0.8 (0.5)	33.5	32.0	34.6
	Other processed foods^c^	> 2.5 (3.3)	1.5–2.5 (2.0)	< 1.5 (1.0)	34.0	35.6	30.4
	Boiled potatoes	> 0.7 (0.9)	0.5–0.7 (0.6)	< 0.5 (0.3)	40.8	35.9	23.3
	Milk, 3% fat	> 0.9 (1.0)	0.1–0.9 (0.3)	0	10.5	7.8	81.7
	Sugar and honey	> 0.9 (2.0)	0.1–0.9 (0.3)	0	32.9	14.8	52.3
	Soft drinks	> 0.2 (1.0)	0.1–0.2 (0.3)	0	22.7	16.9	60.3

Abbreviations: eADI, empirical Anti-inflammatory Diet Index; hsCRP, high sensitivity C-reactive protein

^a^ Included: wholegrain bread, macaroni, spaghetti, and rice

^b^ Points; maximum four points: one point one for each - using olive oil for cooking, using olive oil in homemade dressing, using canola oil for cooking, and using canola oil in homemade dressing

^c^ Included: chips/popcorn/cheese puffs, French fries, fried potatoes, buns/cakes, biscuits/wafers/rusks, gateau/confection, candy, ice cream, lingonberry jam, other jam, fruit canned fools/soups, fruit juices, pea soup (majority ready-to-eat), pancakes (majority ready-to-eat), and pizza (often ready-to-eat)

In the Replication group (*n* = 2,216), the median eADI-17 score was 9 (range: 2–16) which was similar to the Discovery group, where the median was 8.5 (range: 2–16). In the Replication group, the Spearman *Rs* between the eADI-17 and the compound inflammatory indicator was -0.30. The correlations between eADI-17 and hsCRP, IL-6, TNF-R1, and TNF-R2 were as follows: -0.17, -0.23, -0.28, and -0.26, respectively. Spearman *Rs* of individual eADI-17 components (servings/day) versus total eADI-17 ranged from -0.36 for soft drinks to 0.53 for vegetables, and versus the compound inflammatory indicator ranged from 0.12 for soft drinks to -0.18 for nuts (Table 2). Similar results were observed in the Discovery group (Table 2).

**Table 2:** Spearman correlation coefficients of the empirical Anti-inflammatory diet index (eADI) eADI-17 and individual components of the eADI with the compound inflammatory factor^a^ in men with hsCRP < 20 mg/L

		Discovery group *n* = 2,216		Replication group *n* = 2,216	
		eADI-17	Compound inflammatory factor^a^	eADI-17	Compound inflammatory factor^a^
**eADI-17** ^**b**^		**--**	-0.31	--	-0.30
**Components of the eADI**					
**Foods with anti-inflammatory potential**					
	Vegetables	0.54	-0.17	0.53	-0.15
	Olive oil/canola oil	0.52	-0.15	0.51	-0.14
	Nuts	0.50	-0.19	0.51	-0.18
	Seeds	0.50	-0.14	0.46	-0.17
	Legumes	0.45	-0.14	0.45	-0.12
	Wine	0.43	-0.18	0.40	-0.13
	Muesli	0.39	-0.13	0.40	-0.17
	Chicken	0.37	-0.11	0.39	-0.14
	Eggs	0.32	-0.09	0.37	-0.11
	Wholegrains^c^	0.22	-0.05	0.23	-0.03
	Coffee	0.21	-0.10	0.18	-0.11
**Foods with pro-inflammatory potential**					
	Soft drinks	-0.39	0.13	-0.36	0.12
	Sugar and honey	-0.32	0.10	-0.32	0.10
	Milk, 3% fat	-0.23	0.10	-0.22	0.07
	Boiled potatoes	-0.22	0.07	-0.24	0.03
	Processed meat	-0.17	0.10	-0.17	0.08
	Other processed foods^d^	-0.19	0.07	-0.22	0.06

Abbreviations: eADI, empirical Anti-inflammatory Diet Index; hsCRP, high sensitivity C-reactive protein

^a^ Compound inflammatory factor is the 1st factor obtained using principal component analysis based on four inflammatory biomarkers (hsCRP, interleukin 6, tumor necrosis factor receptor 1, and tumor necrosis factor receptor 2

^b^ Spearman correlation coefficients between the eADI-17 and hsCRP, IL-6, TNF-R1, and TNF-R2 were -0.16, -0.26, -0.28, and -0.27, respectively, in the Discovery group, and -0.17, -0.23, -0.28, and -0.26, respectively, in the Replication group

^c^ Included: wholegrain bread, macaroni, spaghetti, and rice

^d^ Included: chips/popcorn/cheese puffs, French fries, fried potatoes, buns/cakes, biscuits/wafers/rusks, gateau/confection, candy, ice cream, lingonberry jam, other jam, fruit canned fools/soups, fruit juices, pea soup (majority ready-to-eat), pancakes (majority ready-to-eat), and pizza (often ready-to-eat)

Although hsCRP was included alongside other biomarkers in the development of the eADI-17, fewer components of the index showed significant correlations with hsCRP (11 and 12 out of 17 in the Discovery and Replication groups, respectively) than with IL-6 (16 and 13), TNF-R1 (16 and 16) and TNF-R2 (16 and 16) (Supplementary Table S4).

### Characteristics of the participants by eADI-17 categories

Age-standardized characteristics of men across eADI-17 categories in the Replication group were similar to those observed in the Discovery group (Supplementary Table 5S). In the Replication group, men with the highest eADI-17 (≥ 12.5 scores; a diet rich in anti-inflammatory foods and low in pro-inflammatory foods) had a 55.0% lower median plasma hsCRP concentration, 15.2% lower median IL-6, 8.2% lower TNF-R1, and 9.5% lower TNF-R2 levels compared to those with the lowest eADI-17 (≤ 6 scores; diet poor in anti-inflammatory and high in pro-inflammatory foods). Men with the lowest eADI-17 compared to those with the highest were older, less likely to have a university education, less likely to be physically active (≥ 20 min/day), and more likely to be current smokers. Moreover, men with the lowest versus those with the highest eADI-17 were more likely to have used NSAID within the past year and had higher BMI and sagittal measurements (reflecting visceral fat accumulation). As expected, higher eADI-17 scores were associated with higher consumption of foods with anti-inflammatory potential and lower consumption of foods with pro-inflammatory potential.

### Associations between eADI-17 and inflammatory parameters

In the Replication group, similar to the Discovery group, statistically significant inverse associations were observed between the eADI-17 and relative concentrations of hsCRP, IL-6, TNF-R1, and TNF-R2 (Table 3). In the Replication group, each increment by 4.5-point eADI-17 (corresponding to 2 SD) was associated with concentrations that were 12% lower for hsCRP, 6% lower for IL-6, 8% lower for TNF-R1, and 9% lower for TNF-R2.

**Table 3:** Relative concentration of hsCRP, IL-6, TNF-R1 and TNF-R2 by categories of the empirical Anti-inflammatory diet Index-17 (eADI-17) in the Discovery (*n* = 2,216) and the Replication group (*n* = 2,216) men with hsCRP < 20 mg/L; multiple linear regression, β-coefficients (95% confidence intervals)

	Relative concentrations by categories of eADI-17 (β-coefficients with 95% CI)^a^					Per 4.5-point (2SD) increase in eADI^b^	*P*-trend^c^
eADI-17, range (median)	2–6 (5.5/5)^d^	6.5–8 (7.5)	8.5–9.5 (9)	10–12 (10.5/11)^d^	12.5–16 (13)		
***hsCRP***							
Discovery group	1.00 (Ref.)	0.90 (0.79–1.01)	0.87 (0.76–0.99)	0.81 (0.71–0.92)	0.71 (0.60–0.85)	0.85 (0.79–0.92)	< 0.001
Replication group	1.00 (Ref.)	0.95 (0.84–1.07)	0.89 (0.78–1.01)	0.86 (0.75–0.98)	0.72 (0.60–0.86)	0.88 (0.82–0.95)	0.001
***IL-6***							
Discovery group	1.00 (Ref.)	0.91 (0.85–0.98)	0.84 (0.78–0.91)	0.86 (0.79–0.92)	0.75 (0.68–0.83)	0.89 (0.85–0.93)	< 0.001
Replication group	1.00 (Ref.)	0.90 (0.84–0.97)	0.86 (0.80–0.93)	0.90 (0.83–0.97)	0.86 (0.77–0.95)	0.94 (0.90–0.98)	0.005
***TNF-R1***							
Discovery group	1.00 (Ref.)	0.94 (0.91–0.98)	0.92 (0.88–0.95)	0.89 (0.86–0.92)	0.83 (0.79–0.88)	0.91 (0.89–0.93)	< 0.001
Replication group	1.00 (Ref.)	0.95 (0.91–0.98)	0.92 (0.89–0.96)	0.90 (0.86–0.93)	0.86 (0.81–0.91)	0.92 (0.90–0.94)	< 0.001
***TNF-R2***							
Discovery group	1.00 (Ref.)	0.92 (0.88–0.97)	0.89 (0.85–0.94)	0.86 (0.82–0.91)	0.81 (0.76–0.87)	0.91 (0.88–0.93)	< 0.001
Replication group	1.00 (Ref.)	0.92 (0.89–0.97)	0.92 (0.88–0.96)	0.88 (0.84–0.92)	0.83 (0.78–0.88)	0.91 (0.88–0.93)	< 0.001

Abbreviations: CI, confidence interval; eADI, empirical Anti-inflammatory Diet Index; hsCRP, high-sensitivity C-reactive protein; IL-6, interleukin 6; TNF-R1, tumor necrosis factor receptor 1; TNF-R2, tumor necrosis factor receptor 2

^a^ β-coefficients with 95% CI adjusted for age (years, continuous), education (primary, high school, university), walking/cycling (< 20, 20–40, 40–60, > 60 min/day), smoking status (current, former, never), NSAID use during last year (no, yes), statin use during last year (no, yes), cortisone use during last year (no, yes), antibiotic users during last year (no, < 3 months, 3–6 months, 6–12 months), sleeping (≤ 7, > 7 h/day), body mass index (< 18.5, 18.5–24.9, 25-29.9, ≥ 30 kg/m^2^), sagittal measure (cm, quintiles), Charlson comorbidity index (scores, continuous), and total energy intake (kcal/day, quintiles)

^b^ 4.5-point increase in eADI is equal to 2 standard deviations in the Discovery group

^c^ The *P*-trend was calculated including the eADI-17 as a continuous variable adjusted for covariates

hsCRP values were back-transformed (2^x^) because of log2 transformed hsCRP concentration before analysis; IL-6, TNF-R1 and TNF-R2 concentrations were presented in log2 scale NPX (Normalized Protein eXpression), the values were back-transformed (2^NPX^) to linear NPX scale

^d^ Median in the Discovery group / median in the Replication group

### Subgroup analysis by eADI-17 categories

To explore the hypothesis of whether the eADI-17 has the potential to reduce hsCRP concentrations across different hsCRP categories (< 15, < 10, <5, and < 3 mg/L) and to lower inflammation within subgroups defined by factors with inflammatory potential (such as age, smoking status, sagittal abdominal diameter, or presence of chronic diseases), subgroup analyses were conducted (Table 4). To increase statistical power, these analyses were conducted among all men (after combining the Discovery and Replication groups).

**Table 4:** Relative concentration of hsCRP by categories of the empirical Anti-inflammatory diet Index-17 (eADI-17) stratified by age, smoking status, sagittal measure, and Charlson’s comorbidity index among 4,432 men (63–94 years old) with hsCRP < 20 mg/L; multiple linear regression β-coefficients (95% confidence intervals)^a^

	Relative concentrations by eADI-17 (β-coefficients with 95% CI)^a^						Per 4.5-point (2SD) increase in eADI^b^	*P*-trend^c^
eADI-17, range (median)	2–6 (5.5)	6.5–8 (7.5)		8.5–9.5 (9)	10–12 (10.5)	12.5–16 (13)		
***By hsCRP concentration***								
<15 mg/L, *n* = 4,*380*	1.00 (Ref.)	0.94 (0.86–1.03)		0.90 (0.83–0.99)	0.87 (0.80–0.95)	0.75 (0.67–0.85)	0.88 (0.84–0.93)	< 0.001
<10 mg/L, *n* = 4,*296*	1.00 (Ref.)	0.97 (0.90–1.06)		0.93 (0.85–1.01)	0.90 (0.82–0.98)	0.77 (0.69–0.87)	0.89 (0.85–0.94)	< 0.001
<5 mg/L, *n* = 3,*903*	1.00 (Ref.)	0.99 (0.91–1.06)		0.97 (0.90–1.05)	0.91 (0.84–0.99)	0.81 (0.73–0.91)	0.91 (0.86–0.95)	< 0.001
<3 mg/L, *n* = 3,*374*	1.00 (Ref.)	0.96 (0.80–1.04)		0.97 (0.89–1.04)	0.91 (0.84–0.99)	0.84 (0.75–0.93)	0.92 (0.88–0.97)	0.001
***Age***								
63–74 years, *n* = 2,*352*	1.00 (Ref.)	0.97 (0.84–1.10)		0.91 (0.80–1.05)	0.85 (0.74–0.97)	0.74 (0.63–0.87)	0.86 (0.80–0.93)	< 0.001
75–84 years, *n* = 1,*742*	1.00 (Ref.)	0.85 (0.75–0.97)		0.86 (0.75–0.99)	0.85 (0.73–0.97)	0.72 (0.57–0.91)	0.89 (0.81–0.97)	0.013
85–94 years, *n* = 338	1.00 (Ref.)	0.93 (0.73–1.17)		0.70 (0.51–0.97)	0.72 (0.51–1.02)	--^d^	0.76 (0.60–0.96)	0.021
***Smoking status***								
Ever smokers, *n* = 2,*551*	1.00 (Ref.)	0.90 (0.80–1.01)		0.85 (0.76–0.97)	0.84 (0.74–0.94)	0.68 (0.57–0.81)	0.85 (0.80–0.92)	< 0.001
Never smokers, *n* = 1,*881*	1.00 (Ref.)	0.93 (0.82–1.06)		0.90 (0.78–1.03)	0.82 (0.72–0.94)	0.76 (0.63–0.91)	0.88 (0.81–0.95)	0.002
***Sagittal measure***								
≤ median (22.0 cm), *n* = 2,*259*	1.00 (Ref.)	0.95 (0.83–1.07)		0.88 (0.77–1.01)	0.85 (0.74–0.97)	0.67 (0.57–0.79)	0.84 (0.78–0.91)	< 0.001
>median (22.0 cm), *n* = 2,*173*	1.00 (Ref.)	0.89 (0.80–1.00)		0.87 (0.77–0.98)	0.82 (0.72–0.93)	0.84 (0.69–1.03)	0.90 (0.83–0.97)	0.010
***Charlson comorbidity index***								
0 score, *n* = 2,*830*	1.00 (Ref.)	0.93 (0.82–1.04)		0.85 (0.76–0.96)	0.81 (0.72–0.91)	0.73 (0.62–0.85)	0.85 (0.79–0.91)	< 0.001
≥ 1 scores, *n* = 1,*602*	1.00 (Ref.)	0.90 (0.78–1.02)		0.91 (0.79–1.05)	0.89 (0.77–1.03)	0.69 (0.55–0.85)	0.91 (0.83–0.99)	0.035

^a^ Adjusted for age (years, continuous), education (primary, high school, university), walking/cycling (< 20, 20–40, 40–60, > 60 min/day), smoking status (current, former, never), NSAID use during last year (no, yes), statin use during last year (no, yes), cortisone use during last year (no, yes), antibiotic use during last year (no, < 3 months, 3–6 months, 6–12 months), sleeping (≤ 7, > 7 h/day), body mass index (< 18.5, 18.5–24.9, 25-29.9, ≥ 30 kg/m^2^), sagittal (cm, quintiles), Charlson comorbidity index (scores, continuous), and total energy intake (kcal/day, quintiles)

^b^4.5-point increase in eADI is equal to 2 standard deviations

^c^ The *P*-trend was calculated including the eADI-17 as a continuous variable adjusted for covariates

^d^ Due to the limited number of men in the highest category of eADI-17, the *P*-trend was calculated for the eADI-17 in the range of 0–12

All values were back-transformed (2^x^) because of log2 transformed hsCRP concentration before analysis

Higher adherence to the anti-inflammatory diet pattern was associated with lower hsCRP, even in men with relatively low hsCRP concentrations (< 3 mg/L) (Table 4). This shows that the association was not solely dependent on relatively higher levels of inflammation and that the diet has the potential to reduce inflammation also in people with relatively low inflammatory parameters. Likewise, stratification of analyses by age, smoking status, sagittal measure, and Charlson’s comorbidity index indicated that the association between the score and hsCRP concentration was robust. In all stratified subgroups, a higher anti-inflammatory diet potential as measured by the eADI-17 was associated with the lower concentration of hsCRP (Table 4).

### Results for adapted version eADI to population-based cohorts’ data

In a final assessment, we adapted the eADI (based on the 145-item FFQ) to match that of the shorter 96-item FFQ1997, which has been used to collect food consumption data in the large population-based cohorts of Swedish men and women, including nearly 100,000 participants [13, 14]. Since the FFQ1997 did not include questions on “seeds” or “legumes”, the eADI had to be based on 15 instead of 17 food groups, omitting these two groups of foods to create eADI-15. Results for the adapted score were essentially similar to those obtained for eADI-17 (Supplementary Table 6S). In the Replication group, each increment by 4.5-point eADI-15 was associated with concentrations that were 13% lower for hsCRP, 7% lower for IL-6, 9% lower for TNF-R1, and 10% lower for TNF-R2. The eADI-15 was statistically significantly correlated with hsCRP, IL-6, TNF-R1, and TNF-R2. In the Replication group, the Spearman *Rs* were -0.16, -0.22, -0.28, and -0.26, respectively.

## Discussion

In a discovery dataset, which consisted of 50% of 4,432 men that completed an FFQ and had four biomarkers of low-grade inflammation measured in plasma, we used PCA to derive an inflammatory variable, termed the “compound inflammatory factor.” Using this factor, Lasso regression was performed to identify key dietary components associated with pro- and anti-inflammatory status. Based on these findings, we developed the eADI-17 score, which was calculated for each individual by categorizing dietary intake into tertiles of pro- and anti-inflammatory foods. A higher eADI-17 score reflected a higher intake of anti-inflammatory foods relative to pro-inflammatory foods, whereas a lower score indicated the opposite. To validate the eADI-17 score, we tested its predictive ability in an independent dataset, demonstrating a significant association with systemic inflammation, as measured by four inflammatory biomarkers: hsCRP, IL-6, TNF-R1, and TNF-R2. In summary, the eADI-17 score, which can be easily calculated based on dietary intake tertiles, constitutes a meaningful dietary index to predict low-grade chronic systemic inflammation.

In this study, the Spearman correlation coefficient between eADI-17 and inflammatory biomarkers varied from -0.17 for hsCRP to -0.28 for TNF-R1. Compared to the eADI-17, the previously developed anti-inflammatory diet index (AIDI-20) [11] showed a similar correlation with hsCRP (Spearman correlation coefficient of -0.19), but slightly stronger correlations than the previously developed pro-inflammatory diet index (EDII) [10]. The Spearman correlations between EDII and hsCRP, IL-6, and TNF-R2 were 0.21, 0.19, and 0.15, respectively (an opposite pro-inflammatory index) [10].

The foods identified to be important for the anti-inflammatory potential in the discovery dataset are consistent with the current knowledge on healthy diets. Food groups with an anti-inflammatory potential, including vegetables, muesli (primarily based on whole grains), olive and canola oil, nuts, legumes, and seeds, are key components of the Mediterranean diet, which has been reported to reduce inflammation [27, 28]. A meta-analysis of randomized controlled trials demonstrated that a Mediterranean diet led to reduced low-grade inflammation as measured by the lowering to IL-6 and CRP concentrations [27, 28]. Furthermore, meta-analyses of randomized controlled trials examining individual food products indicate that fruits and vegetables [29], olive oil [30], and legumes [31, 32] have the potential to reduce inflammation (mainly measured by CRP concentration). However, the effects of nuts [33] and seeds [34–36] as well as wholegrains [37] in reducing low-grade inflammation are not conclusive and require further investigation.

Foods classified as pro-inflammatory in the eADI-17, such as processed red meat, other processed foods, sugar, and soft drinks, are typical components of the Western dietary pattern, which has been linked to promoting inflammation [38]. It was shown that, unlike the Mediterranean diet, the Western dietary pattern has a pro-inflammatory impact, negatively affecting both the immune system and gut microbiota [39].

Even though numerous studies on alcohol consumption in relation to risk and mortality from CVD have been conducted, the results are still not clear [40–42]. The World Health Organization and other authorities emphasize that there is no safe amount of alcohol consumption, particularly concerning cancer risk [43–45]. It should be emphasized that in our study, most of the studied men (78.4%) consumed low or moderate amounts of alcoholic beverages (≤ 7 servings/week), with wine being the only alcoholic beverage included in the eADI-17. Importantly, non-consumers of alcohol are never advised to start drinking. It has been hypothesized that the observed statistically significant inverse correlations between wine consumption and inflammatory biomarkers may be attributed to the polyphenols in wine, which have known anti-inflammatory properties and contribute to reduced systemic inflammation [46].

As the eADI is based on a diet pattern with the potential to reduce inflammation, it can be assumed that poultry and eggs may reflect pro-healthy behaviors of replacing red meat, while boiled potatoes may be indicative of poorer dietary choices. However, based on current evidence, although boiled potatoes have a high glycemic index, there is no conclusive evidence demonstrating their harmful effects on health [47].

The strengths of the eADI-17 include its development based on a strong methodological foundation, using four individual inflammatory parameters. Unlike previously proposed indices, the eADI-17 is based on the portion size (servings per week) of foods with anti-inflammatory or pro-inflammatory potential, making it user-friendly. Another strength of the index is that it was developed in the large sub-cohort of the population-based COSM^CS^ with extensive data collection on many significant covariates, including smoking status, physical activity, NSAID use, statin use, and antibiotic use. The FFQ used to collect data about food consumption had relatively high validity [18]; however, misclassification of food consumption was inevitable. Our study used the National Swedish Prescribed Drug Register to collect information about prescribed drugs such as antibiotics, NSAIDs, statins, and cortisone during the year before blood collection. However, it is plausible that some medications may have been omitted, e.g., intravenous antibiotics administered in hospitals and NSAIDs bought over the counter. Although the multivariable-adjusted linear regression models were adjusted for multiple confounders, unmeasured or residual confounding cannot be disregarded. Another limitation of the study is its cross-sectional design, which precludes the establishment of causal relationships. However, our findings are consistent with those reported in meta-analyses of randomized controlled trials that examined the effects of specific foods (such as fruits, vegetables, olive oil, and legumes [29–32]) and certain dietary patterns (including the Mediterranean diet and the Western dietary pattern [27, 28, 38, 39]) on inflammatory status. The study population of elderly Swedish men might limit the generalizability of the index to other populations with different age groups, sex, and food habits. Furthermore, although inflammatory biomarkers are widely used, their clinical relevance and ability to mirror the inflammatory state may be questioned [48]. However, a prospective study with a 10-year follow-up indicated that IL-6 and TNFα are suitable for assessing low-grade inflammation in older adults and the elderly and may be used to predict mortality [49]. CRP is an acute-phase reactant protein that has both pro-inflammatory and anti-inflammatory properties. Elevated CRP may be associated both with acute and chronic conditions, and these can be infectious or non-infectious in etiology. Thus, CRP is a non-specific marker of inflammation [16]. In our study, although hsCRP (along with other biomarkers) was used to create the compound inflammation indicator for identifying of diet–inflammation associations, 5 out of the 17 food groups in the Discovery group and 4 in the Replication group did not show a significant correlation with hsCRP. In contrast, only 1 and 2 food groups in the Discovery and Replication groups, respectively, were not significantly correlated with IL-6, and just 1 food group lacked significant correlation with both TNF-R1 and TNF-R2. These findings suggest that, although CRP is commonly used to assess the relationship between diet and systemic inflammation, other biomarkers may be more sensitive indicators of diet-related inflammatory responses. Therefore, multiple inflammatory biomarkers should be used to investigate such associations because the use of a single marker may be insufficient and may limit the accuracy and comprehensiveness of the results.

Before the eADI-17 can be widely used in personalized nutrition, it should be validated in other populations in different age groups of men and women, and in populations with other food habits. Developing a simple, user-friendly anti-inflammatory diet index has significant public health and clinical implications, as diet-related low-grade inflammation may contribute to the development of chronic diseases linked to inflammation. Since the index is based on portion sizes of food consumption, it could potentially be an easy tool for personalized dietary advice. However, its use in dietary counseling would need further validation before implementation. The cut-offs for eADI components established during the development process can be directly used to identify patients at risk of low-grade inflammation with similar dietary habits; however, patients with different dietary patterns may require revision. Furthermore, the index could also be used at the population level to assess the anti-inflammatory potential of diets, predict low-grade inflammation, and evaluate the risk of developing systemic inflammation diseases.

## Conclusion

In summary, the eADI-17 represents an empirically developed, user-friendly index for assessing the anti-inflammatory potential of diet, which can be applied at the individual level. Its ability to predict low-grade inflammation should be tested in other populations. This index has the potential to further refine future dietary guidelines and to be used in personalized nutrition.

## Electronic supplementary material

Below is the link to the electronic supplementary material.

Supplementary Material 1

## Data Availability

The data and the analytical program are stored on a highly secure institutional server under the supervision of Alicja Wolk (PI). Investigators may apply to access the study’s deidentified data through contact with the PI.
