# Correction: Development of empirical anti-inflammatory diet index: a cross-sectional study

**DOI:** 10.1186/s12937-025-01208-3

**Published:** 2025-08-22

**Authors:** Joanna Kaluza, Lisa Hellerström, Daniel Kaluza, Abbas Chabok, Agneta Åkesson, Karl Michaëlsson, Alicja Wolk

**Affiliations:** 1 Unit of Cardiovascular and Nutritional Epidemiology, Institute of Environmental Medicine, Karolinska Institutet, Stockholm, SE-171 77 Sweden; 2 Department of Human Nutrition, Warsaw University of Life Sciences – SGGW, Warsaw, 02-776 Poland; 3 Centre for Clinical Research, Västerås Hospital, Uppsala University, Västerås, 721 89 Sweden; 4 Institute of Informatics, University of Warsaw, Warsaw, 02-097 Poland; 5 Division of Surgery, Danderyd University Hospital, Stockholm, 182 88 Sweden; 6 Medical Epidemiology, Department of Surgical Sciences, Uppsala University, Uppsala, SE-751 85 Sweden

**Correction: Nutr J 24**,** 97 (2025)**

**https://doi.org/10.1186/s12937-025-01165-x**

Following publication of the original article [1], the author reported that Table 4 contained incorrect values in the row presenting results By hsCRP concentration for participants with hsCRP < 15 mg/L.

Incorect Table 4

**Table 4** Relative concentration of HsCRP by categories of the empirical Anti-inflammatory diet Index-17 (eADI-17) stratified by age, smoking status, sagittal measure, and charlson’s comorbidity index among 4,432 men (63–94 years old) with HsCRP < 20 mg/L; multiple linear regression β-coefficients (95% confidence intervals)^a^

**Table:** 

	Relative concentrations by eADI-17 (β-coefficients with 95% CI)^a^					Per 4.5-point (2SD) increase in eADI^b^	*P*-trend^c^
eADI-17, range (median)	2–6 (5.5)	6.5–8 (7.5)	8.5–9.5 (9)	10–12 (10.5)	12.5–16 (13)		
***By hsCRP concentration***							
<15 mg/L, *n* = 4,*380*	1.00 (Ref.)	0.94 (0.86–1.03)	0.87 (0.80–0.95)	0.75 (0.67–0.85)	0.88 (0.84–0.93)		< 0.001

Correct Table 4

**Table 4:** Relative concentration of hsCRP by categories of the empirical Anti-inflammatory Diet Index-17 (eADI-17) stratified by age, smoking status, sagittal measure, and Charlson’s comorbidity index among 4,432 men (63-94 years old) with hsCRP <20 mg/L; Multiple linear regression β-coefficients (95% confidence intervals)^a^

	Relative concentrations by eADI-17 (β-coefficients with 95% CI)^a^					Per 4.5-point (2SD) increase in eADI^b^	*P*-trend^c^
eADI-17, range (median)	2–6 (5.5)	6.5–8 (7.5)	8.5–9.5 (9)	10–12 (10.5)	12.5–16 (13)		
***By hsCRP concentration***							
<15 mg/L, *n* = 4,*380*	1.00 (Ref.)	0.94 (0.86–1.03)	0.90 (0.83–0.99)	0.87 (0.80–0.95)	0.75 (0.67–0.85)	0.88 (0.84–0.93)	< 0.001

The original article has been updated.
