# Supplementary material for: Development of empirical anti-inflammatory diet index: a cross-sectional study
Source: Nutr J. 2025 Jun 23;24:97. doi: 10.1186/s12937-025-01165-x (PMC12183828; doi:10.1186/s12937-025-01165-x)
Supplement: Supplementary file 1 — Supplementary Material 1 [file 12937_2025_1165_MOESM1_ESM.docx]

**Development of Empirical Anti-inflammatory Diet Index: A Cross-Sectional Study**

Joanna Kaluza^1,2,3^*, Lisa Hellerström^1^, Daniel Kaluza^4^,  Abbas Chabok^3,5^, Agneta Åkesson^1^, Karl Michaëlsson^6^, Alicja Wolk^1,6^

**Affiliations:**

^1^ Unit of Cardiovascular and Nutritional Epidemiology, Institute of Environmental Medicine, Karolinska Institutet, SE-171 77 Stockholm, Sweden

^2^ Department of Human Nutrition, Warsaw University of Life Sciences–SGGW, 02-776 Warsaw, Poland

^3^ Centre for Clinical Research, Västerås Hospital, Uppsala University, 721 89 Västerås, Sweden

^4^ Institute of Informatics, University of Warsaw, 02-097 Warsaw, Poland

^5^ Division of Surgery, Danderyd University Hospital, 182 88 Stockholm, Sweden

^6^ Medical Epidemiology, Department of Surgical Sciences, Uppsala University, SE-751 85, Uppsala, Sweden

**Supplementary Material**

**Contents**

| **Supplementary Table S1:** Specific foods included in components of the empirical Anti-inflammatory Diet Index -17 (eADI-17) – (based on 145-item FFQ)^a^ and in the eAIDI-15 (based on 96-item FFQ) ……………………………………………………. | 2 |
| --- | --- |
| **Supplementary Table S2:** Stages of the k-fold cross-validation feature selection procedure with hyperparametrization used in this study to identify foods with anti-inflammatory and pro-inflammatory potential ………………………………………… | 7 |
| **Supplementary Table S3:** Cross-validation of selected food components **(bold style)** included in the empirical Anti-Inflammatory Diet Index (eADI) based on Lasso regression coefficients across all food groups for the compound inflammatory indicator (Discovery group of 2,216 men) ………………………………………………………. | 8 |
| **Supplementary Table S4:** Spearman correlations between food components of the empirical Anti-inflammatory Diet Index-17 (eADI-17) and four individual inflammatory biomarkers in men with hsCRP<20 mg/L ……………………………… | 10 |
| **Supplementary Table S5:** Age-standardized characteristics of participants (men 63-94 years old with hsCRP <20 mg/L) by categories of the empirical Anti-inflammatory Diet Index-17 (eADI-17) in the Discovery and Replication group …………………… | 12 |
| **Supplementary Table S6:** Relative concentration of hsCRP, IL-6, TNF-R1 and TNF-R2 by categories of the adapted^a^ empirical Anti-inflammatory Diet Index-15 (eADI-15) among men with hsCRP <20 mg/L; multiple linear regression β-coefficients (95% confidence intervals) …………………………………………………………………… | 16 |
| **Supplementary Figure 1S.** Visualization of (A) Spearman correlation coefficients for the first principal component (PC1) versus the second principal component (PC2) for the inflammatory parameters, visualization of (B) correlation loadings of PC1 and PC2 and the inflammatory parameters, and (C) Spearman correlation coefficients between the inflammatory parameters in the Discovery group. …………………………………………….. | 17 |

**Supplementary Table S1.**  Specific foods included in components of the empirical Anti-inflammatory Diet Index -17 (eADI-17) – (based on 145-item FFQ)^a^ and in the eAIDI-15 (based on 96-item FFQ)

|  | **eADI-17 components**  **FFQ 2009 (145 items)** | **eADI-15 components**  **FFQ 1997 (96 items)** |
| --- | --- | --- |
| **Vegetables** | **Carrots**  **Beetroots**  **Lettuce/iceberg**  **Spinach**  **Cabbage**  **Cauliflower**  **Broccoli/brussels sprouts**  **Tomato/tomato juice**  **Pepper**  **Green peas**  **Onion**  **Leek**  **Garlic**  **Mix frozen vegetables**  **Other vegetables**  **Avocado**  **Olives**  **Sweetcorn** | **Carrots**  **Beetroots**  **Lettuce**  **Spinach**  **Cabbage**  **Cauliflower**  **Broccoli/brussels sprouts**  **Tomato/ tomato juice**  **Pepper**  **Green peas**  **Onion/leek**  **Garlic**  **Other/mix vegetables** |
| Fruits (fresh) | Orange/citrus fruits  Apple/pears  Banana  Berries  Other fruits | Orange/citrus fruits  Apple/pear  Banana  Berries  Other fruits |
| Fruits (dry) | Raisins  Prunes (including juice)  Apricots/other dried fruits | *Lack* |
| **Wholegrain bread/ macaroni/spaghetti/**  **rice** | **Spaghetti/macaroni/pasta**  **Wholegrain rice**  **Wholegrain bread**  **Crispbread/hard bread**  **Fiber enriched bread** | **Wholegrain bread**  **Crispbread/hard bread** |
| White bread/ macaroni/spaghetti/rice | Spaghetti/macaroni/pasta  White bread  White rice  Couscous/bulgur | Spaghetti/macaroni/pasta  White bread  Rice |
| **Muesli** | **Muesli** | **Cereals/muesli** |
| Other breakfast cereal | Oatmeal  Gruel/other porridge  Wheat/oat bran  Other breakfast cereal | Oatmeal  Gruel/other porridge  Wheat/oat bran |
| **Potatoes** | **Boiled potatoes**  **Baked/mashed potatoes** | **Cooked potatoes** |
| **Nuts** | **Peanuts**  **Other nuts/almonds** | **Nuts/almonds** |
| **Seeds** | **Sunflower/pumpkin seeds**  **Linseeds**  **Sesame seeds** | *Lack* |
| **Legumes** | **Beans/lentils/chick peas** | *Lack* |
| Herbs and spices | Fresh herbs  Dried herbs  Pepper  Cinnamon | *Lack* |
| Fresh meat | Pork  Beef/veal  Minced meat  Other meat | Pork  Beef/veal  Minced meat |
| **Processed meat** | **Lean sausage**  **Bolonga sausage**  **Other sausage**  **Black pudding/sausage**  **Liver pate**  **Liver pate low-fat**  **Cold cuts (meat toppings e.g. ham/turkey)**  **Cold cuts (sausage toppings e.g. salami)**  **Bacon** | **Sausage**  **Black pudding**  **Liver pate**  **Cold cuts** |
| Organ meat | Liver/kidney | Liver/kidney |
| Fish and seafood | Herring/mackerel  Salmon  Codfish/saithe/plaice/grenadier  Tuna  Pike/perch/bass  Fish fingers  Other fish  Smoked fish  Roe  Caviar  Shellfish | Herring/mackerel  Salmon  Codfish/saithe  Caviar  Shellfish |
| **Chicken/other poultry** | **Chicken/other poultry** | **Chicken/other poultry** |
| **Eggs/omelet** | **Eggs/omelette** | **Eggs/omelette** |
| **Milk (3% fat)** | **Milk (3.0% fat)** | **Milk (3.0% fat)** |
| Milk (0.5/1.5% fat) | Milk (<0.5% fat)  Milk (1.5% fat) | Milk (<0.5% fat)  Milk (1.5% fat) |
| Yogurt/sour milk | Yogurt/sour milk (<0.5% fat)  Sour milk (1.5% fat)  Yogurt/sour milk (3% fat)  Fruit yogurt/sour milk | Sour milk/yogurt (0.5% fat)  Sour milk/yogurt (3.0% fat) |
| Cheese | Hard cheese  Hard cheese, low fat  Cottage cheese  Cream cheese  Cream cheese, low fat  Dessert cheese | Hard cheese (>24% fat)  Low-fat cheese (<17% fat)  Cottage cheese |
| Tea | Black tea  Green tea  Herbal/red tea | Tea |
| **Coffee** | **Filtered coffee**  **Unfiltered coffee** | **Coffee** |
| **Wine** | **Red wine**  **White wine** | **Wine** |
| Beer | Light beer  Classic beer  Strong beer | Light beer  Classic beer  Strong beer |
| Strong alcohol | Liqueur/sherry/fortified wines  Spirits | Liqueur/sherry/fortified wines  Spirits |
| **Soft drinks** | **Coca cola/pepsi**  **Coca cola/pepsi light**  **Other soft drinks/soda**  **Other soft drinks/soda light** | **Soft drinks/soda** |
| **Sugar and honey** | **Sugar**  **Honey** | **Sugar/honey** |
| Chocolate | Chocolate | Chocolate |
| **Other processed foods** | **Chips/popcorn/cheese puffs**  **French fries**  **Fried potatoes**  **Buns/cakes**  **Biscuits/wafers/rusks**  **Gateau/confection**  **Candy**  **Ice cream**  **Lingonberry jam**  **Other jam**  **Fruit canned fools/soups**  **Fruit juices**  **Pea soup (majority ready-to-eat)**  **Pancakes (majority ready-to-eat)**  **Pizza (often ready-to-eat)** | **Chips/popcorn/cheese puffs**  **French potatoes**  **Fried potatoes**  **Buns/cakes**  **Biscuits/wafers**  **Cakes/pastries**  **Candy**  **Ice cream**  **Jam/marmalade/sauce**  **Fruit fool/fruit soup**  **Orange/grapefruit juice**  **Pea soup/beans/lentils**  **Pancakes**  **Soya bean products**  **Pizza** |
| Salad dressing | Salad dressing  Salad dressing, reduced fat  Mayonnaise  Mayonnaise, reduce fat  Double cream  Single cream/sour cream  Crème fraiche  Cream fraiche, reduced fat  Yogurt for cooking, 8-10% fat  Ketchup | Salad dressing  Salad dressing, reduced fat  Mayonnaise  Mayonnaise, reduced fat  Cream  Cream fraiche  Cream fraiche, reduced fat  Ketchup |
| **Olive oil and rapeseed oil** | **Olive oil**  **Rapeseed oil** | **Olive oil**  **Rapeseed oil** |
| Butter (80% fat) | Butter (80% fat) | Butter (80% fat) |
| Bregott (50% butter/50% margarine) | Bregott (50% butter/50% margarine) | Bregott (50% butter/50% margarine) |
| Margarine (80% fat) | Margarine (80% fat) | Margarine (80% fat) |
| Margarine spread (80% fat) | Margarine spread (80% fat) | Margarine spread (80% fat) |
| Margarine spread (40% fat) | Margarine spread (40% fat) | Margarine spread (40% fat) |
| Cooking oil (corn/sunflower/soybean) | Cooking oil (corn/sunflower/soybean) | Cooking oil (corn/sunflower/soybean) |
| Liquid margarine | Liquid margarine | Liquid margarine |

^a^ Of 145 food items 63 were correlated with the compound inflammatory marker and included in the eADI-15;82 food items were not correlated.

Food groups marked grey are the foods included in the eADI.

**Supplementary Table S2.** Stages of the k-fold cross-validation feature selection procedure with hyperparametrization used in this study to identify foods with anti-inflammatory and pro-inflammatory potential

| **K-fold feature selection procedure** |  | **Hyperparameters used in the study** |
| --- | --- | --- |
| ***Stage 1 – Data division*** |  |  |
| Dividing dataset into k data folds equal in size |  | k = 10, dividing the Discovery group (n=2,216) into 10 folds |
| ***Stage 2 – Filtering procedure*** |  |  |
| The feature filtering procedure is repeated k times; At each iteration k - 1 folds are used for training and feature selection; Each iteration results in boolean indicators describing whether a particular feature has been selected |  | The features filtering procedure is repeated 10 times, the 33 food groups were filtered using Lasso regression model with the compound inflammatory indicator as a dependent variable (developed in PCA based on the four individual inflammatory biomarkers – details presented below); Features with non-zero model coefficient are marked as selection in this fold |
| ***Stage 3 – Feature collection*** |  |  |
| The final set of features are those selected in P folds, where P is a hyperparameter of the algorithm |  | P ≥9, thus only the most robust features (food groups), i.e., those that occurred in every or almost every fold, were chosen |

**Supplementary Table S3.** Cross-validation of selected food components **(bold style)** included in the empirical Anti-Inflammatory Diet Index (eADI) based on Lasso regression coefficients across all food groups for the compound inflammatory indicator (Discovery group of 2,216 men)

|  | Lasso regression coefficients from the 10-fold feature procedure | | | | | | | | | | | SUM |
| --- | --- | --- | --- | --- | --- | --- | --- | --- | --- | --- | --- | --- |
|  | 1 | 2 | 3 | 4 | 5 | 6 | 7 | 8 | 9 | 10 | Mean (SD) |  |
| **Wholegrain bread/ macaroni/spaghetti /rice** | **-0.008** | **-0.004** | **-0.015** | **-0.007** | **-0.007** | **--** | **-0.008** | **-0.016** | **-0.007** | **-0.014** | **-0.010 (0.004)** | **9** |
| White bread/ macaroni/spaghetti/rice | -- | -- | -- | -- | -- | -- | -- | -- | -- | -- | -- | 0 |
| **Muesli** | **-0.094** | **-0.070** | **-0.132** | **-0.133** | **-0.076** | **-0.143** | **-0.054** | **-0.073** | **-0.088** | **-0.066** | **-0.093 (0.032)** | **10** |
| Other breakfast cereal | -- | -- | -- | -- | -- | -- | -- | -- | -- | -- |  | 0 |
| **Vegetables** | **-0.029** | **-0.034** | **-0.040** | **-0.018** | **-0.037** | **-0.043** | **-0.026** | **-0.025** | **-0.016** | **-0.024** | **-0.029 (0.009)** | **10** |
| Fruits | -- | -- | -- | -- | -- | -- | -- | -- | -- | -- | -- | 0 |
| Dry fruits | -- | -- | -- | -- | -- | -- | -- | -- | -- | -- | -- | 0 |
| **Boiled potatoes** | **0.091** | **0.082** | **0.110** | **0.090** | **0.067** | **0.092** | **0.029** | **0.089** | **0.062** | **0.080** | **0.079 (0.022)** | **10** |
| **Nuts** | **-0.079** | **-0.058** | **-0.134** | **-0.123** | **-0.095** | **-0.144** | **-0.142** | **-0.093** | **-0.105** | **-0.118** | **-0.109 (0.028)** | **10** |
| **Seeds** | **-0.054** | **-0.081** | **-0.063** | **-0.046** | **-0.096** | **-0.044** | **-0.075** | **-0.073** | **-0.055** | **-0.089** | **-0.068 (0.018)** | **10** |
| **Legumes** | **-0.085** | **-0.121** | **-0.021** | **-0.266** | **-0.063** | **-0.351** | **-0.128** | **-0.058** | **-0.193** | **-0.162** | **-0.145 (0.102)** | **10** |
| Herbs/spices | -0.074 | -0.117 | -- | -0.066 | -0.088 | -- | -0.073 | -0.098 | -0.089 | -0.050 | -- | 8 |
| **Olive/canola** | **-0.068** | **-0.052** | **-0.075** | **-0.070** | **-0.059** | **-0.061** | **-0.054** | **-0.056** | **-0.062** | **-0.053** | **-0.061 (0.008)** | **10** |
| Tea | -- | -- | -- | -- | -- | -- | -- | -- | -- | -- | -- | 0 |
| **Coffee** | **-0.024** | **-0.020** | **-0.020** | **-0.059** | **-0.023** | **-0.016** | **-0.023** | **-0.023** | **-0.021** | **-0.017** | **-0.025 (0.012)** | **10** |
| Fresh meat | -- | -0.025 | -0.114 | -- | -- | -0.002 | -- | -0.036 | -- | -- | -- | 4 |
| Organ meat | 0.066 | 0.117 | 0.253 | 0.095 | -- | 0.580 | 1.088 | -- | 0.277 | 0.011 | -- | 8 |
| **Processed meat** | **0.106** | **0.069** | **0.093** | **0.080** | **0.099** | **0.090** | **0.095** | **0.107** | **0.090** | **0.088** | **0.092 (0.011)** | **10** |
| **Other processed foods** | **0.027** | **0.047** | **0.026** | **0.044** | **0.030** | **0.036** | **0.022** | **0.027** | **0.037** | **0.024** | **0.032 (0.009)** | **10** |
| Fish/seafood | -- | -- | -- | -- | -- | -- | -- | -- | -- | -- | -- | 0 |
| **Chicken** | **-0.415** | **--** | **-0.342** | **-0.331** | **-0.299** | **-0.244** | **-0.478** | **-0.337** | **-0.447** | **-0.409** | **-0.367 (0.075)** | **9** |
| **Eggs**  **3% fat milk** | **-0.053** | **-0.086** | **-0.043** | **-0.069** | **-0.037** | **-0.116** | **-0.096** | **-0.115** | **-0.064** | **-0.071** | **-0.075 (0.028)** | **10** |
|  | **0.167** | **0.167** | **0.175** | **0.192** | **0.184** | **0.155** | **0.194** | **0.176** | **0.174** | **0.180** | **0.176 (0.012)** | **10** |
| 0.5% fat/1.5% fat milk | -- | -- | -- | -- | -- | -- | -- | -- | -- | -- | -- | 0 |
| Yogurt/sour milk | -- | -- | -- | -- | -- | -- | -- | -- | -- | -- | -- | 0 |
| Cheese | -- | -0.005 | -0.001 | -- | -- | -0.013 | -- | -0.006 | -- | -- | -- | 4 |
| **Sugar/honey** | **0.012** | **0.025** | **0.021** | **0.016** | **0.017** | **0.014** | **0.012** | **0.024** | **0.014** | **0.017** | **0.017 (0.005)** | **10** |
| Chocolate | -- | -- | -- | -- | -- | -- | -- | -- | -- | -- | -- | 0 |
| **Soft drinks** | **0.126** | **0.100** | **0.145** | **0.154** | **0.142** | **0.169** | **0.121** | **0.147** | **0.127** | **0.123** | **0.135 (0.020)** | **10** |
| **Wine** | **-0.217** | **-0.266** | **-0.195** | **-0.311** | **-0.187** | **-0.210** | **-0.232** | **-0.201** | **-0.220** | **-0.258** | **-0.230 (0.038)** | **10** |
| Beer | -0.092 | -0.015 | -0.005 | **--** | -0.023 | -0.093 | -0.048 | -- | -- | -0.027 | -- | 7 |
| Strong alcohol | -- | -- | -0.084 | -- | -- | -- | -- | -- | -- | -- | -- | 1 |
| Salad dressing | -- | -- | -- | -- | -- | -- | -- | -- | -- | -- | -- | 0 |

In each iteration, Lasso regression was trained on 9 out of 10 folds using the compound inflammation indicator (the 1^st^ factor in PCA) as a dependent variable. Positive coefficients indicate pro-inflammatory patterns, and negative correspond to anti-inflammatory patterns. The "Sum" column denotes the number of non-zero coefficients for a particular food groups across the folds. Only those food groups were selected for eADI for which, in the Discovery group, Lasso regression obtained non-zero coefficients in ≥9 folds and for which Spearman correlation coefficients were statistically significant for at least 3 out of 4 inflammatory parameters.

**Supplementary Table S4.** Spearman correlations between food components of the empirical Anti-inflammatory Diet Index-17 (eADI-17) and four individual inflammatory biomarkers in men with hsCRP <20 mg/L

|  | | **Discovery group**  **n = 2,216** | | | | | | | | | |  | | **Replication group**  **n = 2,216** | | | | | | | | | |
| --- | --- | --- | --- | --- | --- | --- | --- | --- | --- | --- | --- | --- | --- | --- | --- | --- | --- | --- | --- | --- | --- | --- | --- |
|  | | | **hsCRP** | **IL-6** | | **TNF-R1** | | **TNF-R2** | | |  | | **hsCRP** | | | | **IL-6** | **TNF-R1** | | | **TNF-R2** | |  |
| **Foods with anti-inflammatory potential** | | | | |  | | |  |  | | | | | |  | | | |  | | |  | |
|  | Vegetables | | -0.10 | -0.14 | | -0.15 | | -0.15 | | |  | | -0.11 | | | | -0.14 | -0.11 | | | -0.12 | |  |
|  | Olive oil/canola oil | | -0.09 | -0.12 | | -0.13 | | -0.12 | | |  | | -0.06 | | | | -0.10 | -0.13 | | | -0.12 | |  |
|  | Nuts | | -0.09 | -0.16 | | -0.16 | | -0.17 | | |  | | -0.10 | | | | -0.15 | -0.15 | | | -0.15 | |  |
|  | Seeds | | -0.11 | -0.14 | | -0.09 | | -0.09 | | |  | | -0.12 | | | | -0.16 | -0.13 | | | -0.12 | |  |
|  | Legumes | | -0.08 | -0.12 | | -0.10 | | -0.11 | | |  | | -0.05 | | | | -0.09 | -0.11 | | | -0.11 | |  |
|  | Wine | | -0.09 | -0.12 | | -0.19 | | -0.17 | | |  | | -0.04 | | | | -0.07 | -0.14 | | | -0.13 | |  |
|  | Muesli | | -0.09 | -0.14 | | -0.09 | | -0.07 | | |  | | -0.13 | | | | -0.15 | -0.14 | | | -0.12 | |  |
|  | Chicken | | ns^a^ | -0.12 | | -0.10 | | -0.11 | | |  | | -0.06 | | | | -0.10 | -0.13 | | | -0.13 | |  |
|  | Eggs | | ns | -0.08 | | -0.11 | | -0.10 | | |  | | ns | | | | -0.07 | -0.10 | | | -0.11 | |  |
|  | Wholegrain bread/ macaroni/spaghetti /rice | | -0.05 | -0.08 | | ns^a^ | | ns | | |  | | -0.05 | | | | -0.04 | ns | | | ns | |  |
| **Foods with pro-inflammatory potential** | | | | | | |  | | |  | | | | | |  | | | |  | | |  |
|  | Soft drinks | | 0.07 | 0.09 | | 0.13 | | 0.12 | | |  | | 0.08 | | | | 0.09 | 0.12 | | | 0.09 | |  |
|  | Sugar and honey | | 0.07 | 0.06 | | 0.09 | | 0.09 | | |  | | ns | | | | ns | 0.11 | | | 0.11 | |  |
|  | Milk, 3% fat | | ns | 0.05 | | 0.11 | | 0.10 | | |  | | 0.06 | | | | 0.06 | 0.05 | | | 0.05 | |  |
|  | Processed meat | | 0.07 | 0.09 | | 0.07 | | 0.06 | | |  | | 0.05 | | | | 0.05 | 0.08 | | | 0.07 | |  |
|  | Other processed foods^b^ | | ns | 0.04 | | 0.08 | | 0.09 | | |  | | ns | | | | ns | 0.12 | | | 0.11 | |  |
|  | Boiled potatoes | | ns | ns | | 0.08 | | 0.08 | | |  | | ns | | | | ns | 0.05 | | | 0.04 | |  |

hsCRP = High sensitivity C-reactive protein; IL-6 = Interleukin 6; TNF-R1 = Tumor necrosis factor receptor 1; TNF-R2 = Tumor necrosis factor receptor 2;

^a^ ns – statistically non-significant, *P*-*value* >0.5 for Spearman correlation.

^b^ Included: chips/popcorn/cheese puffs, French fries, fried potatoes, buns/cakes, biscuits/wafers/rusks, gateau/confection, candy, ice cream, lingonberry jam, other jam, fruit canned fools/soups, fruit juices, pea soup (majority ready-to-eat), pancakes (majority ready-to-eat), pizza (often ready-to-eat).

**Supplementary Table S5.** Age-standardized characteristics of participants (men 63-94 years old with hsCRP <20 mg/L) by categories of the empirical Anti-inflammatory Diet Index-17 (eADI-17) in the Discovery and Replication group

|  | **Discovery group *n = 2,216*** | | | | | | | | | |  | **Replication group *n = 2,216*** | | | | |
| --- | --- | --- | --- | --- | --- | --- | --- | --- | --- | --- | --- | --- | --- | --- | --- | --- |
|  | Categories of eADI-17, range (median) | | | | | | | | | |  | Categories of eADI-17, range (median) | | | | |
|  | 2 – 6 (5.5) | | 6.5 – 8 (7.5) | | 8.5 – 9.5 (9) | | 10 – 12 (10.5) | | | 12.5 – 16 (13) |  | 2 – 6 (5) | 6.5 – 8 (7.5) | 8.5 – 9.5 (9) | 10 – 12 (11) | 12.5 – 16 (13) |
| N | 341 | | 622 | | 492 | | 594 | | | 167 |  | 310 | 601 | 548 | 592 | 165 |
| Age, years | 77.4 ± 6.5 ^a^ | | 75.0 ± 6.2 | | 73.8 ± 5.3 | | 73.1 ± 5.5 | | | 71.1 ± 4.7 |  | 77.5 ± 6.5 | 75.4 ± 6.3 | 73.7 ± 5.7 | 72.6 ± 5.2 | 70.9 ± 4.9 |
| Plasma hsCRP, mg/L; median (range) | 1.9 (0.1 – 19.0) | | 1.6 (0.1 – 17.0) | | 1.5 (0.1 – 19.0) | | 1.3 (0.1 – 17.0) | | | 1.1 (0.1 – 14.0) |  | 2.0 (0.1 – 19.0) | 1.6 (0.1 – 19.0) | 1.5 (0.1 – 18.0) | 1.3 (0.1 – 17.0) | 0.9 (0.1 – 14.0) |
| IL-6, NPX; median (range) | 4.6 (3.1 – 10.0) | | 4.4 (2.7 – 9.3) | | 4.2 (2.1 – 7.1) | | 4.1 (2.6 – 10.1) | | | 3.9 (2.8 – 6.1) |  | 4.6 (2.9 – 8.1) | 4.4 (2.8 – 9.8) | 4.2 (2.6 – 7.9) | 4.1 (1.1 – 9.4) | 3.9 (2.8 – 10.1) |
| TNF-R1, NPX; median (range) | 4.9 (3.9 – 7.1) | | 4.7 (3.5 – 6.5) | | 4.7 (3.4 – 6.7) | | 4.6 (3.2 – 6.6) | | | 4.4 (3.6 – 5.5) |  | 4.9 (3.8 – 6.9) | 4.7 (3.7 – 6.6) | 4.7 (3.5 – 6.3) | 4.6 (2.9 – 6.6) | 4.5 (3.5 – 5.8) |
| TNF-R2, NPX; median (range) | 4.2 (3.2 – 10.7) | | 4.0 (2.8 – 6.5) | | 4.0 (2.8 – 10.1) | | 3.9 (2.5 – 10.1) | | | 3.8 (2.7 – 4.9) |  | 4.2 (3.3 – 6.4) | 4.0 (3.1 – 6.0) | 4.0 (3.0 – 10.0) | 3.9 (2.2 – 5.8) | 3.8 (2.7 – 5.0) |
| Education; %  Primary  High school  University | 34.3  56.0  9.7 | | 28.2  57.7  14.1 | | 21.1  56.7  22.2 | | 15.4  54.6  30.0 | | | 11.2  53.7  35.0 |  | 33.0  57.0  10.0 | 26.1  58.2  15.7 | 25.1  56.0  18.8 | 14.8  54.3  30.9 | 9.6  46.6  43.8 |
| Walking/cycling, min/day; %  < 20  20-40  40-60  > 60 | 27.7  37.9  16.3  18.1 | | 29.7  37.0  20.1  13.2 | | 20.4  39.6  20.8  19.2 | | 14.4  40.9  26.4  18.3 | | | 15.5  47.7  20.4  16.4 |  | 32.3  36.1  15.9  15.6 | 28.1  38.8  20.8  12.3 | 23.7  43.6  15.8  16.8 | 18.9  38.5  23.8  18.8 | 7.9  40.6  33.8  17.8 |
| Smoking status; %  Current  Former  Never | 11.0  41.6  47.4 | | 8.3  46.9  44.8 | | 5.3  49.0  45.8 | | 2.7  47.3  50.0 | | | 2.7  48.4  48.9 |  | 11.1  45.1  43.8 | 9.3  47.4  43.3 | 7.3  49.7  43.0 | 3.3  53.3  43.4 | 4.4  48.4  47.3 |
| NSAID use within last 1 year; %^b^ | 16.0 | | 12.0 | | 11.1 | | 15.5 | | | 8.8 |  | 12.9 | 14.7 | 13.3 | 11.8 | 10.7 |
| Cortisone use within last 1 year; %^b^ | 8.8 | | 5.4 | | 5.9 | | 4.8 | | | 10.2 |  | 6.9 | 5.5 | 6.3 | 6.3 | 3.9 |
| Statin use within last 1 year; %^b^ | 33.7 | | 40.7 | | 40.1 | | 38.3 | | | 43.3 |  | 37.6 | 38.9 | 38.3 | 35.7 | 39.9 |
| Antibiotic users during the last year; %^b^ | |  | | | |  | | |  | |  |  |  |  |  |  |
| No | 81.1 | | 81.4 | | 81.3 | | 80.1 | | | 72.6 |  | 75.1 | 81.1 | 80.9 | 77.9 | 82.7 |
| <3 months from blood collection | 4.2 | | 6.1 | | 6.5 | | 6.1 | | | 12.5 |  | 5.9 | 5.4 | 6.4 | 4.9 | 4.6 |
| 3-6 months from blood collection | 6.8 | | 4.9 | | 4.9 | | 5.0 | | | 9.1 |  | 6.3 | 4.5 | 5.0 | 7.9 | 2.7 |
| 6-12 months from blood collection | 7.9 | | 7.6 | | 8.1 | | 8.8 | | | 5.8 |  | 12.7 | 8.9 | 7.7 | 9.3 | 10.0 |
| Sleeping ≤ 7 hours/day; % | 72.5 | | 67.7 | | 68.4 | | 69.8 | | | 70.3 |  | 67.9 | 65.5 | 70.3 | 72.3 | 68.2 |
| Body mass index, kg/m^2^ | 27.0 ± 4.0 | | 26.9 ± 3.9 | | 26.7 ± 3.7 | | 26.1 ± 3.5 | | | 26.1 ± 2.9 |  | 26.5 ± 3.8 | 26.9 ± 3.8 | 26.8 ± 3.6 | 26.4 ± 3.5 | 25.0 ± 3.2 |
| Sagittal, cm | 23.2 ± 3.1 | | 23.0 ± 3.3 | | 22.7 ± 3.2 | | 22.0 ± 3.1 | | | 21.8 ± 2.7 |  | 22.8 ± 3.4 | 22.8 ± 3.1 | 22.7 ± 3.1 | 22.4 ± 3.1 | 21.0 ± 2.7 |
| Charlson comorbidity index, scores; %  0  1  2  ≥ 3 | 62.7  16.9  12.0  8.4 | | 59.4  17.8  14.2  8.6 | | 63.8  16.2  12.7  7.3 | | 65.3  20.2  9.4  5.2 | | | 64.7  18.9  9.5  6.9 |  | 59.9  19.2  13.8  7.0 | 62.6  17.6  12.0  7.7 | 63.5  17.5  11.1  7.8 | 69.6  15.5  10.1  4.9 | 67.0  15.9  12.6  4.4 |
| Total energy intake, kcal/day | 2408 ± 656 | | 2315 ± 644 | | 2487 ± 662 | | 2608 ± 687 | | | 2598 ± 606 |  | 2340 ± 703 | 2319 ± 647 | 2440 ± 697 | 2520 ± 683 | 2783 ± 678 |
| *Foods with anti-inflammatory potential* |  | | | *Servings, mean ± SD* | | | |  | | |  | *Servings, mean ± SD* | | | | |
| Per day |  | |  | |  | |  | | |  |  |  |  |  |  |  |
| Wholegrains^c^ | 3.0 ± 2.1 | | 3.5 ± 2.2 | | 4.0 ± 2.2 | | 4.3 ± 2.6 | | | 4.3 ± 2.0 |  | 2.7 ± 2.0 | 3.4 ± 2.2 | 3.9 ± 2.4 | 4.1 ± 2.3 | 4.5 ± 2.4 |
| Vegetables | 2.0 ± 1.4 | | 2.6 ± 1.5 | | 3.2 ± 1.8 | | 4.3 ± 2.2 | | | 5.2 ± 1.9 |  | 1.9 ± 1.3 | 2.6 ± 1.6 | 3.4 ± 1.8 | 4.2 ± 2.0 | 5.1 ± 1.8 |
| Coffee | 2.3 ± 1.5 | | 2.6 ± 1.6 | | 2.8 ± 1.6 | | 2.9 ± 1.4 | | | 3.2 ± 1.5 |  | 2.2 ± 1.5 | 2.5 ± 1.5 | 2.8 ± 1.7 | 2.8 ± 1.6 | 2.9 ± 1.5 |
| Olive oil and canola oil^d^ | 0.6 ± 0.8 | | 1.0 ± 1.0 | | 1.5 ± 1.1 | | 2.0 ± 1.0 | | | 2.4 ± 0.9 |  | 0.6 ± 0.8 | 1.1 ± 1.0 | 1.6 ± 1.1 | 2.0 ± 1.0 | 2.4 ± 1.0 |
| Per week |  | |  | |  | |  | | |  |  |  |  |  |  |  |
| Chicken | 0.6 ± 0.6 | | 0.8 ± 0.7 | | 1.0 ± 0.8 | | 1.2 ± 0.8 | | | 1.5 ± 0.9 |  | 0.6 ± 0.6 | 0.8 ± 0.6 | 1.0 ± 0.7 | 1.3 ± 1.0 | 1.4 ± 0.8 |
| Eggs | 1.2 ± 1.4 | | 1.7 ± 1.8 | | 2.1 ± 2.2 | | 2.4 ± 2.4 | | | 3.4 ± 2.5 |  | 1.2 ± 1.9 | 1.7 ± 2.0 | 2.1 ± 2.2 | 2.6 ± 2.3 | 3.7 ± 2.5 |
| Muesli | 0.5 ± 1.7 | | 1.2 ± 2.3 | | 2.0 ± 3.0 | | 2.8 ± 3.2 | | | 4.1 ± 3.5 |  | 0.5 ± 1.4 | 1.2 ± 2.4 | 1.9 ± 2.9 | 2.8 ± 3.2 | 4.2 ± 3.2 |
| Nuts | 0.5 ± 1.0 | | 0.8 ± 1.7 | | 1.5 ± 3.2 | | 2.2 ± 2.9 | | | 3.5 ± 3.0 |  | 0.4 ± 1.1 | 0.8 ± 1.6 | 1.2 ± 1.8 | 2.3 ± 3.3 | 4.0 ± 3.3 |
| Seeds | 0.1 ± 0.8 | | 0.5 ± 2.1 | | 1.2 ± 3.3 | | 3.3 ± 5.5 | | | 6.0 ± 6.6 |  | 0.2 ± 1.2 | 0.6 ± 3.3 | 1.3 ± 3.9 | 2.8 ± 5.0 | 6.4 ± 7.0 |
| Legumes | 0.1 ± 0.3 | | 0.2 ± 0.5 | | 0.4 ± 0.6 | | 0.7 ± 1.0 | | | 1.1 ± 2.0 |  | 0.1 ± 0.5 | 0.2 ± 0.5 | 0.4 ± 0.7 | 0.7 ± 1.0 | 1.4 ± 1.4 |
| Wine | 0.6 ± 1.1 | | 1.1 ± 1.6 | | 1.7 ± 1.8 | | 2.3 ± 2.0 | | | 2.8 ± 1.9 |  | 0.4 ± 0.9 | 1.2 ± 1.7 | 1.5 ± 1.9 | 2.2 ± 2.2 | 3.2 ± 2.1 |
| *Foods with pro-inflammatory potential* |  | | | *Servings, mean ± SD* | | | |  | | |  | *Servings, mean ± SD* | | | | |
| Per day |  | |  | |  | |  | | |  |  |  |  |  |  |  |
| Processed meat | 1.6 ± 1.2 | | 1.4 ± 1.0 | | 1.4 ± 1.0 | | 1.3 ± 1.1 | | | 0.9 ± 0.7 |  | 1.5 ± 1.2 | 1.4 ± 1.2 | 1.3 ± 1.1 | 1.2 ± 0.9 | 1.0 ± 0.9 |
| Other processed foods^e^ | 2.5 ± 1.4 | | 2.3 ± 1.3 | | 2.2 ± 1.2 | | 2.1 ± 1.1 | | | 1.5 ± 0.8 |  | 2.6 ± 1.5 | 2.2 ± 1.1 | 2.1 ± 1.1 | 2.0 ± 1.1 | 1.6 ± 1.0 |
| Boiled potatoes | 0.8 ± 0.4 | | 0.7 ± 0.4 | | 0.6 ± 0.4 | | 0.6 ± 0.4 | | | 0.5 ± 0.4 |  | 0.8 ± 0.5 | 0.7 ± 0.4 | 0.6 ± 0.4 | 0.6 ± 0.3 | 0.5 ± 0.3 |
| Milk, 3% fat | 0.5 ± 0.8 | | 0.2 ± 0.5 | | 0.1 ± 0.4 | | 0.1 ± 0.4 | | | 0.04 ± 0.25 |  | 0.5 ± 1.0 | 0.2 ± 0.7 | 0.2 ± 0.6 | 0.1 ± 0.3 | 0.03 ± 0.16 |
| Sugar and honey | 2.1 ± 2.4 | | 1.1 ± 1.8 | | 0.8 ± 1.7 | | 0.4 ± 1.0 | | | 0.4 ± 1.1 |  | 2.0 ± 2.3 | 1.2 ± 2.0 | 0.8 ± 1.5 | 0.5 ± 1.3 | 0.2 ± 0.6 |
| Soft drinks | 0.8 ± 1.1 | | 0.4 ± 0.7 | | 0.2 ± 0.6 | | 0.1 ± 0.3 | | | 0.03 ± 0.17 |  | 0.9 ± 1.4 | 0.4 ± 0.7 | 0.3 ± 0.6 | 0.2 ± 0.5 | 0.07 ± 0.38 |

Abbreviations: BMI, body mass index; eADI, empirical Anti-inflammatory Diet Index; hsCRP, high sensitivity C-reactive protein; IL-6, interleukin 6; NPX, normalized protein expression values (arbitrary units on a log2 scale provided by Olink proteomics); NSAID, nonsteroidal anti-inflammatory drug; TNF-R1, tumor necrosis factor receptor 1; TNF-R2, tumor necrosis factor receptor 2.

^a^ Mean ± SD.

^b^ Based on prescribed medicines from the Swedish National Prescribed Drug Register within one year prior to blood sampling.

^c^ Included: wholegrain bread, macaroni, spaghetti, and rice.

^d^ Points (maximum four points: one point for each – using olive oil for cooking, using olive oil in homemade dressing, using canola oil for cooking, and using canola oil in homemade dressing.

^e^ Include: chips/popcorn/cheese puffs, French fries, fried potatoes, buns/cakes, biscuits/wafers/rusks, gateau/confection, candy, ice cream, lingonberry jam, other jam, fruit canned fools/soups, fruit juices, pea soup (majority ready-to-eat), pancakes (majority ready-to-eat), and pizza (often ready-to-eat).

**Supplementary Table S6.** Relative concentration of hsCRP, IL-6, TNF-R1 and TNF-R2 by categories of the adapted^a^ empirical Anti-inflammatory Diet Index-15 (eADI-15) among men with hsCRP <20 mg/L in the Replication group (n = 2,216); multiple linear regression β-coefficients (95% confidence intervals)^b^

|  | Relative concentrations by eADI-15 (β-coefficients with 95% CI)^b^ | | | | |  | Per 4.5-point increase in eADI^c^ | *P*-trend^d^ |
| --- | --- | --- | --- | --- | --- | --- | --- | --- |
| eADI-15,  range (median) | 2 – 6 (5.5)  n = 372 | 6.5 – 8 (7.5)  n = 712 | 8.5 – 9.5 (9)  n = 602 | 10 – 10.5 (10)  n = 276 | 11 – 14 (11.5)  n = 254 |  |  |  |
| ***hsCRP*** | 1.00 (Ref.) | 0.93 (0.83 – 1.04) | 0.86 (0.77 – 0.97) | 0.84 (0.73 – 0.97) | 0.81 (0.70 – 0.95) |  | 0.87 (0.80 – 0.95) | 0.002 |
| ***IL-6*** | 1.00 (Ref.) | 0.90 (0.84 – 0.95) | 0.86 (0.81 – 0.93) | 0.90 (0.82 – 0.97) | 0.91 (0.84 – 0.99) |  | 0.93 (0.89 – 0.98) | 0.009 |
| ***TNF-R1*** | 1.00 (Ref.) | 0.94 (0.91 – 0.97) | 0.91 (0.88 – 0.95) | 0.88 (0.84 – 0.92) | 0.87 (0.83 – 0.91) |  | 0.91 (0.88 – 0.93) | <0.001 |
| ***TNF-R2*** | 1.00 (Ref.) | 0.92 (0.89 – 0.96) | 0.9 (0.88 – 0.95) | 0.86 (0.82 – 0.91) | 0.85 (0.80 – 0.90) |  | 0.90 (0.88 – 0.93) | <0.001 |

Abbreviations: CI, confidence interval; eADI, empirical Anti-inflammatory Diet Index; hsCRP, high-sensitivity C-reactive protein; IL-6, interleukin 6; TNF-R1, tumor necrosis factor receptor 1; TNF-R2, tumor necrosis factor receptor 2.

^a^ The eADI-17 has been adapted to the shorter FFQ1997 which was used to collect data about food consumption in the large population-based cohorts – the COSM and the SMC.

^b^ Adjusted for age (years, continuous), education (primary, high school, university), walking/cycling (<20, 20-40, 40-60, >60 min/day), smoking status (current, former, never), NSAID use during last year (no, yes), statin use during last year (no, yes), cortisone use during last year (no, yes), antibiotic users during last year (no, <3 months, 3-6 months, 6-12 months), sleeping (≤ 7, >7 hours/day), body mass index (<18.5, 18.5-24.9, 25-29.9, ≥30 kg/m^2^), sagittal (cm, quintiles), Charlson comorbidity index (scores, continuous), and total energy intake (kcal/day, quintiles).

^c^ 4.5-point for increase as for eADI-15.

^d^ The *P*-trend was calculated including the eADI-15 as a continuous variable adjusted for covariates.

hsCRP values were back-transformed (2^x^) because of log2 transformed hsCRP concentration before analysis; IL-6, TNF-R1 and TNF-R2 concentrations were presented in log2 scale NPX (Normalized Protein eXpression), the values were back-transformed (2^NPX^) to linear NPX scale.

| **(A)** | 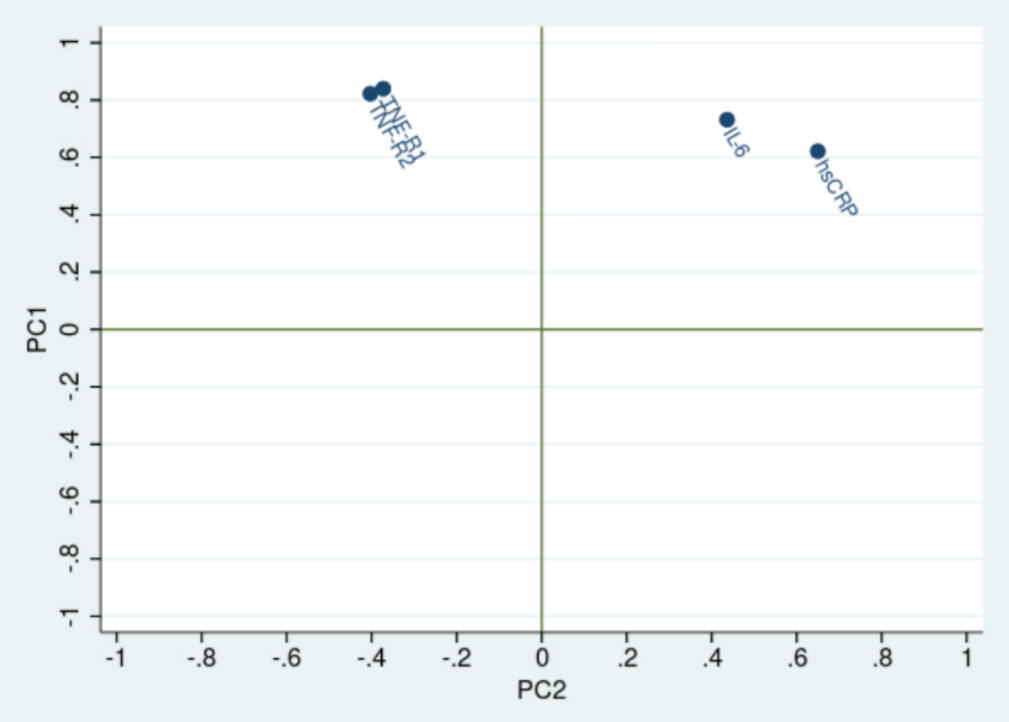 | **Spearman correlation coefficients**  PC1 vs hsCRP = 0.62  PC1 vs IL-6 = 0.73  PC1 vs TNF-R1 = 0.84  PC1 vs TNF-R2 = 0.82  PC2 vs hsCRP = 0.65  PC2 vs IL-6 = 0.44  PC2 vs TNF-R1 = -0.37  PC2 vs TNF-R2 = -0.40 |
| --- | --- | --- |
|  |  |  |
| **(B)** | 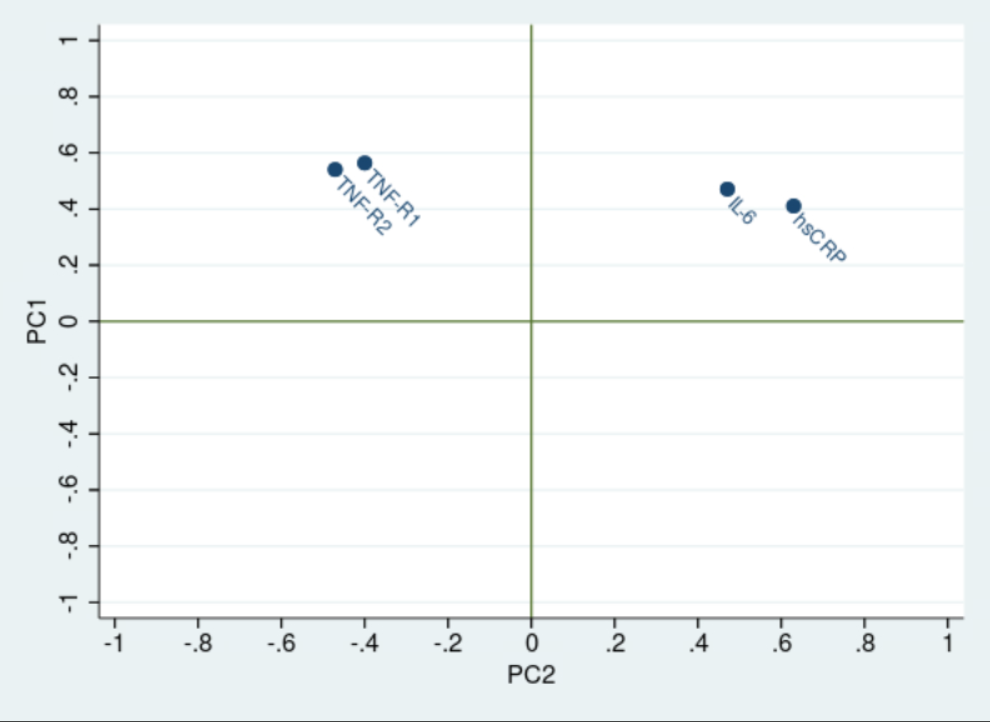 | **Correlation loadings**  PC1 vs hsCRP = 0.41  PC1 vs IL-6 = 0.47  PC1 vs TNF-R1 = 0.56  PC1 vs TNF-R2 = 0.55  PC2 vs hsCRP = 0.63  PC2 vs IL-6 = 0.47  PC2 vs TNF-R1 = -0.40  PC2 vs TNF-R2 = -0.47 |
|  |  |  |
| **(C)** | \| **Spearman correlation coefficients between the inflammatory parameters** \| \| \| \| \| --- \| --- \| --- \| --- \| \|  \| hsCRP \| IL-6 \| TNF-R1 \| \| IL-6 \| 0.52 \| -- \| -- \| \| TNF-R1 \| 0.27 \| 0.41 \| -- \| \| TNF-R2 \| 0.25 \| 0.39 \| 0.86 \| |  |

**Supplementary Figure 1S.** Visualization of (A) Spearman correlation coefficients for the first principal component (PC1) versus the second principal component (PC2) for the inflammatory parameters, visualization of (B) correlation loadings of PC1 and PC2 and the inflammatory parameters, and (C) Spearman correlation coefficients between the inflammatory parameters in the Discovery group.

Abbreviations: hsCRP, high-sensitivity C-reactive protein; IL-6, interleukin 6; TNF-R1, tumor necrosis factor receptor 1; TNF-R2, tumor necrosis factor receptor 2.
